# Large language models enable prognostic stratification of cancer patients using real-world clinical notes

**DOI:** 10.1371/journal.pdig.0001546

**Published:** 2026-07-08

**Authors:** Niklas Kiermeyer, Tim Lenfers, Amin Dada, Julian Friedrich, Sameh Khattab, Eric Knop, Jan Egger, Markus Pauly, Andreas Jung, Grégoire Montavon, Jens T. Siveke, Marcel Wiesweg, Stefan Kasper, Ulf P. Neumann, Frederick Klauschen, Sylvia Hartmann, Martin Schuler, Philipp Keyl, Jens Kleesiek, Julius Keyl

**Affiliations:** 1 German Cancer Consortium (DKTK), partner site Munich, a partnership between DKFZ and Ludwig-Maximilians-Universität München (LMU), Munich, Germany; 2 Institute of Pathology, Ludwig-Maximilians-University Munich, Munich, Germany; 3 Institute for Artificial Intelligence in Medicine, University Hospital Essen, Essen, Germany; 4 Department of Statistics, TU Dortmund University, Dortmund, Germany; 5 Research Center Trustworthy Data Science and Security, University Alliance Ruhr, Dortmund, Germany; 6 BIFOLD – Berlin Institute for the Foundations of Learning and Data, Berlin, Germany; 7 Charité – Universitätsmedizin, Berlin, Germany; 8 Department of Medical Oncology, West German Cancer Center, University Hospital Essen, Essen, Germany; 9 Bridge Institute of Experimental Tumor Therapy (BIT) and Division of Solid Tumor Translational Oncology (DKTK), West German Cancer Center, University Hospital Essen, University of Duisburg-Essen, Essen, Germany; 10 National Center for Tumor Diseases (NCT), NCT West, Essen, Germany; 11 German Cancer Consortium (DKTK), Partner Site Essen, a partnership between University Duisburg-Essen and German Cancer Research Centre (DKFZ), Essen, Germany; 12 Department of General, Visceral, Vascular and Transplantation Surgery, University Hospital Essen, Essen, Germany; 13 Institute of Pathology, Charité - Universitätsmedizin Berlin, Corporate Member of Freie Universität Berlin and Humboldt-Universität Berlin, Berlin, Germany; 14 Bavarian Cancer Research Center (BZKF), Munich, Germany; 15 Institute of Pathology, University Hospital Essen, Essen, Germany; Durham University, UNITED KINGDOM OF GREAT BRITAIN AND NORTHERN IRELAND

## Abstract

In medical documentation, vast amounts of unstructured text are generated that are still underutilized in current prognostic models. We investigate the potential of self-hosted large language models (LLM) to extract clinically meaningful, patient-specific information from routine clinical notes for personalized risk stratification in cancer care. We collected real-world medical notes from 2,708 non-small cell lung cancer (NSCLC) patients and 814 colon cancer patients documented before treatment at a large comprehensive cancer center. LLMs extracted key prognostic indicators, including comorbidities, metastatic sites, and qualitative descriptors of patient condition, in a zero-shot manner without prior task-specific training. Integrating these LLM-derived features into machine learning models significantly improved the prediction of overall survival compared to TNM staging alone (C-Index: NSCLC, 0.72 vs 0.64; colon cancer, 0.70 vs 0.59), and surpassed models using text embeddings. Based on the LLM-informed risk scores, patients were stratified into four distinct risk groups, enabling reclassification of 61.4% of NSCLC and 68.3% of colon cancer patients. Analysis of model drivers revealed that LLM-derived factors, such as the physical condition, substantially modulated the prognostic impact of TNM stage. These findings highlight the potential of self-hosted LLM to derive prognostically relevant information from unstructured clinical documentation and support clinical decision-making.

## Author summary

In routine clinical care, medical staff document large amounts of patient information, but only a fraction is captured in structured electronic health records, while much remains in free-text clinical notes. However, most established scoring systems do not use this information and instead rely on a small set of structured variables, such as tumor stage. In this study, we investigated the use of large language models (LLMs) for the extraction of prognostic information from clinical notes without task-specific training. Applying LLMs to the medical records of more than 3,500 patients with lung and colon cancer, we extracted patient characteristics, including mobility impairment, pain, dyspnea, and comorbidities. These features were validated against structured EHR data and expert physician annotations. The LLM-extracted features substantially improved machine learning–based survival prediction and patient stratification beyond conventional measures such as tumor stage. Furthermore, allowing the LLM to derive its own summary score of patient condition provided strong predictors of patient outcome. These findings demonstrate that artificial intelligence can unlock prognostic information from clinical records at scale, supporting more informed and personalized clinical decision-making.

## Introduction

Unstructured medical reports contain a wealth of doctor-curated patient-specific information that is currently not fully exploited for patient stratification. Especially host-specific information, such as the patient’s physical condition and disease-related symptoms, has substantial influence on patient outcomes and should be an integral part of the clinical decision process and cancer research, complementing tumor-specific information. As these data are currently often not assessed in a structured format, clinical notes remain the only source that regularly incorporates this information. However, as current prognostic systems rely on structured data, this data source remains largely untapped in deployed prognostic systems. Even current multimodal models, which aim to incorporate comprehensive patient information and show great promise in cancer treatment guidance and biomarker discovery, mostly neglect the wealth of information hidden in text data [1–7]. This is due to the fact that extracting relevant information from unstructured text was traditionally a labor-intensive and error-prone process, which often required domain experts to manually review and correct the results. Yet, recent advances in natural language processing (NLP) and Large Language Models (LLMs) have largely removed this barrier, enabling the automated extraction of meaningful information from unstructured text data [8–10]. In particular, the rapid pace of research around open-source LLMs such as Meta’s Llama has created an environment where new and improved models are now released almost monthly [11,12]. Moreover, it enables hospitals to self-host these LLMs, which is essential for use in routine clinical practice and in the context of data privacy. However, the clinical implementation of these existing approaches remains limited, and most approaches are applied on small, non-real-world datasets limiting their generalizability [13,14].

Here, we developed an explainable, end-to-end survival-prediction framework to integrate LLM-extracted features and specialized text embeddings on large, real-world cancer cohorts. To this end, we employed Llama 4 Scout and a dedicated embedding model to process EHR notes and discharge reports from 2,708 patients with NSCLC and 814 patients with colon cancer. We investigate whether LLM-based extraction of routine unstructured clinical notes can improve survival prediction and refine patient stratification beyond standard TNM staging. By converting clinical documentation into structured parameters, we aim to uncover outcome heterogeneity that is not captured by conventional prognostic frameworks alone.

## Results

### Cohort description

Our real-world dataset comprised 2,708 patients diagnosed with non-small cell lung cancer (NSCLC) and a validation cohort of 814 patients with colon cancer treated at the University Hospital Essen (NSCLC: 2017–2025, colon cancer: 2010–2025, Fig 1 + Table 1). For all included patients, unstructured text data from EHR notes and discharge reports prior to the start of cancer therapy were collected. In the NSCLC cohort (median age 67.3 years; 41% female), 996 patients experienced the event (death), while 1712 were right-censored. Adenocarcinoma was the most common histology (61.5%), followed by squamous cell carcinoma (29.8%), large cell carcinoma (4.5%), and adenosquamous carcinoma (4.2%). Stage IV disease accounted for 48.6% of cases (Stage I 23.1%, Stage III 19.0%, Stage II 9.4%). In the colon cancer cohort (median age 66.7 years; 48% female), 510 patients experienced the event, and 304 were right-censored. Stage IV disease was present in 57.7% of cases (Stage III 16.6%, Stage II 14.7%, Stage I 11.3%). Both cohorts reflect the subset of patients with at least one pre-treatment clinical text report and successful feature extraction, representing the identical patient population used across all subsequent analyses (see Methods and S1 Fig).

**Table 1:** Characteristics of the patient cohorts. Bracketed values represent median overall survival (OS) time in months and the 95% confidence intervals (CIs).

	NSCLC (n = 2,708)	Colon cancer (n = 814)
**Median Age**	67 (Range: 24–91)	66.0 (Range: 22–97)
**Sex**		
Female	1,135 (41.9%)	392 (48.2%)
Male	1,573 (58.1%)	422 (51.8%)
**Stage**		
I	625 (23.1%) [not reached]	92 (11.3%) [not reached]
II	254 (9.4%) [not reached]	120 (14.7%) [not reached]
III	514 (19.0%) [40.70, 95% CI: 27.62–51.72]	135 (16.6%) [not reached]
IV	1,315 (48.6%) [18.90, 95% CI: 16.87–21.67]	467 (57.4%) [25.05, 95% CI: 20.94–32.38]
**NSCLC Histology**		
Adenocarcinoma	1,666 (61.5%)	–
Squamous Cell Carcinoma	807 (29.8%)	–
Large Cell Carcinoma	122 (4.5%)	–
Adenosquamous Carcinoma	113 (4.2%)	–
**Text availability**		
EHR notes	2,630 (97.1%)	788 (96.8%)
Discharge reports	1,946 (71.9%)	474 (58.2%)

**Fig 1:**
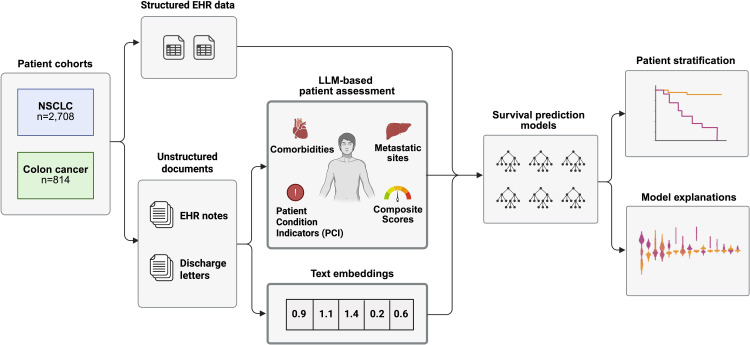
Patient cohorts and study design. Two patient cohorts, non-small cell lung cancer (n = 2,708) and colon cancer (n = 814), were analyzed using structured electronic health record (EHR) data and unstructured clinical documents. The large language model and the text-embedding model were both applied to the same unstructured data: the large language model extracted patient condition indicators (PCIs), comorbidities, metastatic sites, and composite scores, while the text-embedding model generated high-dimensional vector representations. Survival prediction models were compared across different data compositions and used for patient stratification and decision-making analysis.

### Structured extraction of metastatic patterns and comorbidities using LLM

**Fig 2:**
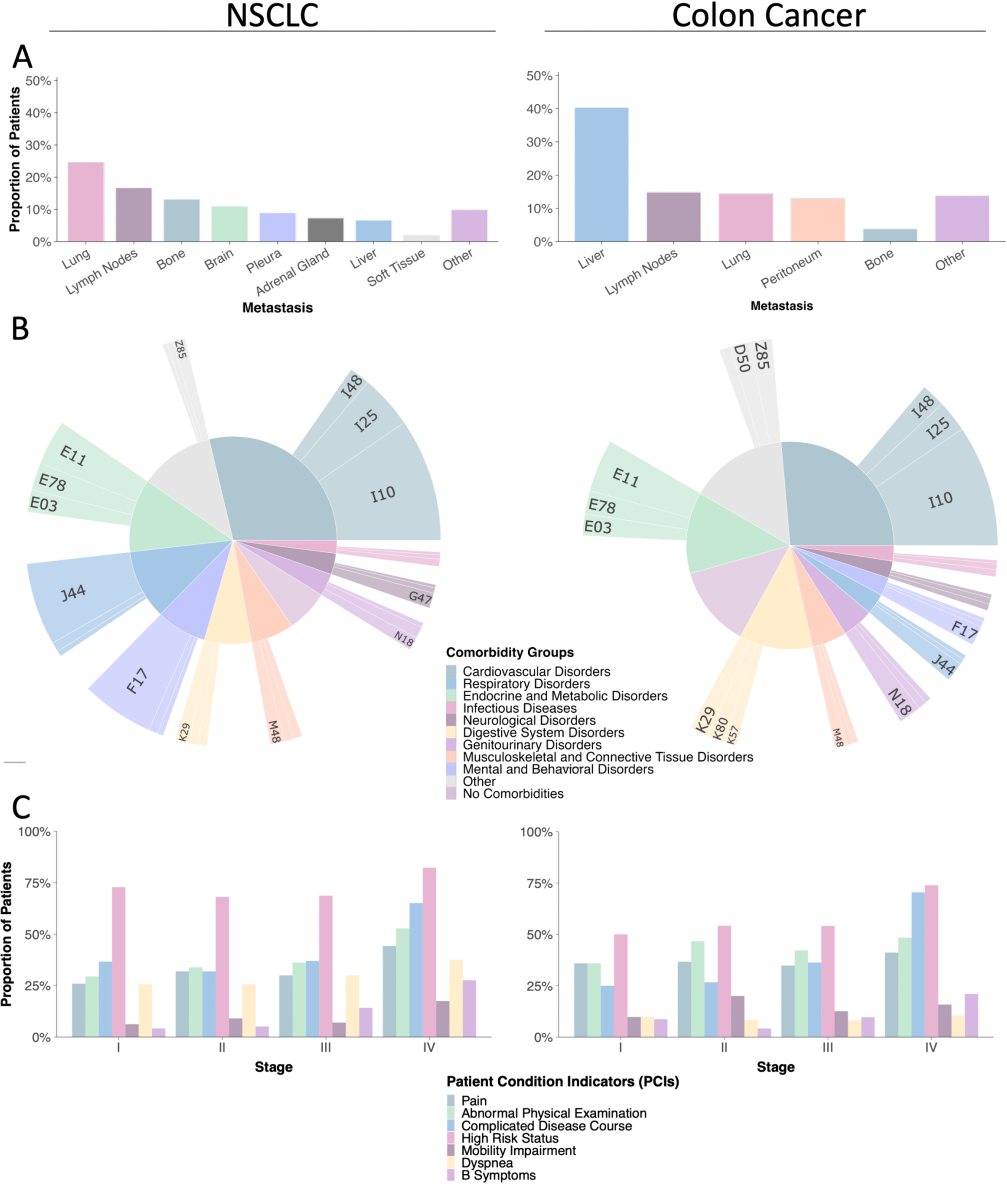
LLM-extracted features in NSCLC and colon cancer cohorts. **A:** Distribution of LLM-extracted metastatic sites in stage 4 NSCLC (left) and colon cancer (right) patients. **B:** Distribution of most frequent comorbidities in NSCLC (left) and colon cancer (right) patients. **C:** Proportion of patients with a positive (True) binary LLM-extracted feature.

To assess whether unstructured text from EHR notes and discharge reports could improve patient stratification beyond TNM staging, we used Llama 4 Scout to automatically extract clinically relevant features prior to the start of treatment at our center. The extracted features comprised metastatic sites, comorbidities (encoded as ICD codes), and seven LLM-inferred patient condition indicators (PCIs): mobility impairment, pain, B-symptoms, high-risk status, dyspnea, complicated disease course, and abnormal physical examination. In addition, the LLM generated two composite scores for each patient: a physical condition score and a survival score inferred only from EHR notes and discharge reports. We observed distinct metastasis patterns in the stage 4 patients of our two cohorts. In NSCLC patients, the most frequently LLM-extracted metastatic sites were the lungs (25%), lymph nodes (17%), and bones (13%) (Fig 2A). In contrast, LLM-extractions for colon cancer patients included predominantly liver metastases (40%), followed by lymph nodes and lung metastases. Extraction quality of metastases was validated on a subset of stage IV patients for which structured information was available in our database (NSCLC: 968, colon cancer: 404; S2 Fig). In addition, the seven LLM-extracted patient condition indicators were validated against expert annotations on a subset of 50 patients per cohort, showing high overall extraction accuracy (macro-average F1: NSCLC 0.73, colon cancer 0.86; S1 and S2 Tables). Performance remained consistent across age, sex, stage, and histology subgroups (S3 and S4 Tables). To further assess the reliability of LLM-based extraction, we evaluated comorbidity extraction against structured ICD-10 codes from the EHR. The majority of LLM-extracted comorbidities were not documented in the structured EHR, reflecting the well-known incompleteness of structured coding rather than extraction errors (S3 Fig). To disentangle possible hallucinations from true extractions missing in the structured EHR, we manually reviewed 30 randomly sampled cases per comorbidity group in both cohorts in which the LLM reported a comorbidity absent from the structured record. This confirmed that hallucinations were rare across organ systems (S3 Fig), with accuracies of 266/269 (98.9%) for NSCLC and 242/251 (96.4%) for Colon. To investigate whether report length influenced the LLM-inferred scores, we computed Spearman correlations between text length (discharge reports, EHR notes, and their combined total) and both scores (S4 Fig). Correlations were weak across both cohorts (r=−0.17 to 0.08).

The LLM-extracted comorbidities also showed cancer-specific patterns after grouping by organ system. As expected, respiratory comorbidities were more frequently extracted in the NSCLC cohort, while disorders of the digestive system were more common in colon cancer patients. However, there were also similarities between both cohorts, as cardiovascular diseases emerged as the most prevalent comorbidity in both cohorts. The top three codes extracted were I10 (hypertension), I25 (ischemic heart disease), and I48 (atrial fibrillation, Fig 2B).

### LLM derive clinically relevant patient condition indicators

Next, we assessed the distribution of LLM-inferred PCI across cohorts and UICC stages. The proportion of patients with a high-risk status increased with disease stage in both cancers, rising from 73% at stage I to 82% by stage IV in NSCLC, and from 50% to 74% in colon cancer (Fig 2C). Similarly, the prevalence of patients with complicated disease courses and abnormal findings on physical examination increased with advancing stages, reflecting higher disease burden and clinical severity at later stages.

Mobility impairment showed a markedly different pattern between cohorts: In NSCLC patients, it was uncommon in early-stage disease (<9% at stages I-II) but increased to 17% by stage IV. Colon cancer patients exhibited mobility impairment of 10–20% in stages I-II, but this did not increase by stage IV (16%). Pain prevalence in NSCLC increased from 26% at stage I to 44% at stage IV, paralleled by a rise in dyspnea (from 26% to 37%). Conversely, colon cancer patients had relatively stable pain (35–41%) and low dyspnea rates (<10%) regardless of stage. B-symptoms were rare (<10%) across stages I-II in both cohorts, but surged in stage IV (28% in NSCLC; 21% in colon cancer), suggesting systemic involvement in advanced disease.

### Prognostic impact of LLM-derived patient condition indicators

To assess the prognostic impact of the LLM-derived PCIs, we performed a multivariate analysis of the NSCLC cohort (Table 2). Tumor stage was the strongest clinical predictor, with Stage IV associated with markedly worse OS compared to Stage I (HR 3.62, p < 0.001). Among LLM-derived PCIs, mobility impairment (HR 1.78, p < 0.001), complicated disease course (HR 1.63, p < 0.001), and abnormal physical examination (HR 1.31, p < 0.001) were independently associated with worse OS. Notably, high-risk status was associated with improved OS (HR 0.71, p < 0.001). In the colon cancer cohort, Stage IV was similarly the strongest predictor (HR 2.84, p < 0.001), while Stage II and III did not reach significance. Among LLM-derived PCIs, mobility impairment (HR 1.79, p < 0.001) and B-symptoms (HR 1.40, p = 0.025) were independently associated with OS.

**Table 2:** Results of multivariate Cox proportional hazards models evaluating the association between LLM-extracted covariates and overall survival. Structured EHR data comprises all fields originally available in structured format, whereas LLM-inferred variables are those derived by the model from unstructured medical documentation.

	NSCLC		Colon Cancer	
Structured EHR Data	HR (95% CI)	P value	HR (95% CI)	P value
Age at Treatment (per 1 SD)	1.11 (1.04-1.18)	**0.001**	1.44 (1.26-1.64)	**<0.001**
Stage II vs I	1.47 (1.04-2.08)	**0.028**	0.61 (0.31-1.21)	0.160
Stage III vs I	2.37 (1.82-3.09)	**<0.001**	0.92 (0.49-1.71)	0.786
Stage IV vs I	3.62 (2.86-4.57)	**<0.001**	2.84 (1.67-4.85)	**<0.001**
Sex (male)	1.36 (1.19-1.55)	**<0.001**	0.93 (0.74-1.17)	0.552
Adenosquamous vs Adenocarcinoma	1.2 (0.88-1.63)	0.253	–	–
Large Cell vs Adenocarcinoma	1.16 (0.84-1.58)	0.368	–	–
Squamous cell vs Adenocarcinoma	1.06 (0.88-1.63)	0.443	–	–
**LLM-Inferred Variables**				
High-Risk Status	0.71 (0.59-0.86)	**<0.001**	1.01 (0.71-1.45)	0.935
Abnormal Physical Examination	1.31 (1.11-1.54)	**<0.001**	1.27 (0.96-1.69)	0.100
Dyspnea	1.07 (0.92-1.24)	0.380	1.12 (0.78-1.62)	0.529
Complicated Disease Course	1.63 (1.37-1.94)	**<0.001**	1.12 (0.8-1.57)	0.518
B-symptoms	1.02 (0.86-1.2)	0.821	1.4 (1.04-1.89)	**0.025**
Pain	1.07 (0.93-1.24)	0.335	0.98 (0.76-1.26)	0.879
Mobility Impairment	1.78 (1.49-2.12)	**<0.001**	1.79 (1.3-2.47)	**<0.001**

Univariate Cox proportional hazards results for individual LLM-derived PCIs are detailed in S5 Table.

### LLM-derived composite scores for physical condition and survival

To enable the LLM to assess patients more comprehensively beyond closely defined markers, we prompted it for two composite scores, a physical condition score (0–100) and a survival score (0–100), based on each patient’s discharge report and EHR notes, resulting in up to four scores per patient. Higher values denote better physical status and greater likelihood of survival. The histograms showed that both physical condition and survival scores are not distributed uniformly for NSCLC and colon cancer, with certain score ranges being notably overrepresented (S5 Fig). In addition, a correlation was observed between both scores and patient condition indicators (S6 Fig). To further validate the LLM-derived physical condition score, we compared it against structured ECOG performance status available for a subset of patients, observing a significant inverse correlation in both cohorts (NSCLC: r=−0.36, p < 0.001, n = 1,657; colon cancer: r = -0.42, p < 0.001, n = 195; S7 Fig). We provide four de-identified text examples to further illustrate LLM-based assessment across clinical contexts (S8 Fig).

In univariate analysis, both LLM-derived composite scores were significantly associated with OS in the NSCLC cohort (physical condition score: HR 0.82, 95% CI: 0.78-0.86, p < 0.001, survival score: HR 0.81, 95% CI: 0.77-0.86; p < 0.001; S6 Table). In multivariate analysis, the physical condition score retained independent prognostic value (p < 0.001).

In univariate analysis of the colon cancer cohort, both the LLM-inferred physical condition score (HR 0.79, 95% CI: 0.72-0.86, p < 0.001) and survival score (HR 0.69, 95% CI: 0.62-0.77, p < 0.001) were significantly associated with OS. Both scores remained independent prognostic markers in multivariate analysis (physical condition: p = 0.02, survival: p = 0.01, S7 Table).

**Fig 3:**
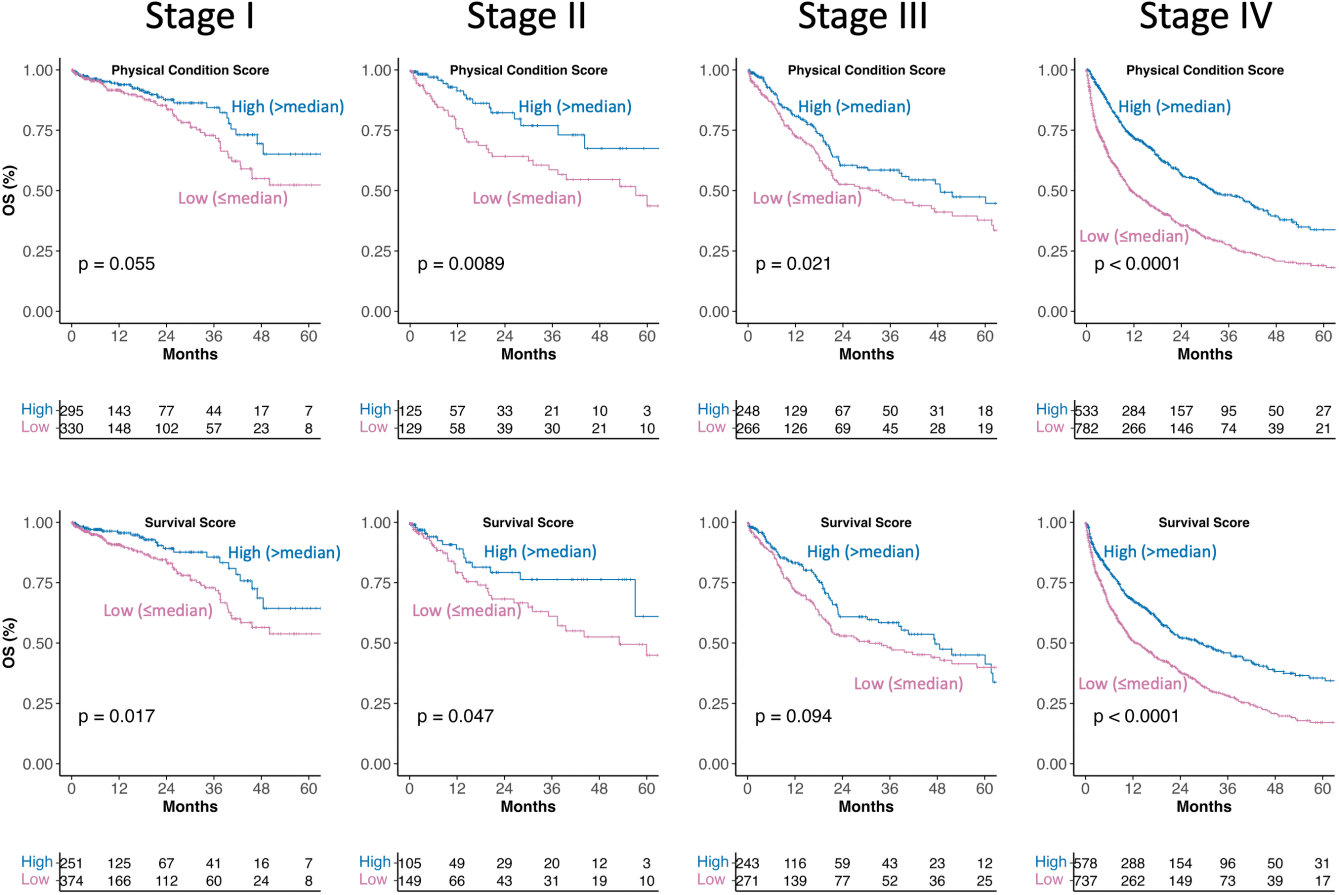
Kaplan-Meier survival curves for NSCLC patients stratified by LLM‑inferred physical condition and survival scores at disease stages I–IV. Patients were dichotomized by the median within their respective stage into high‑score (> median; blue) and low‑score (≤ median; magenta) groups. Survival differences between groups were assessed by the log‑rank test, and corresponding p‑values are indicated in each panel.

Binarizing each LLM-inferred score at its median into “low” and “high” groups yielded a clear stage-wise separation in the NSCLC cohort (Fig 3): all log-rank tests were significant (p < 0.05) except the survival score in stage III and the physical condition score in stage I. The colon cancer cohort showed the same overall pattern, though smaller sample sizes in stages I-III limited statistical power (S9 Fig).

### Comparing LLM-derived information and text embeddings for survival prediction

**Fig 4:**
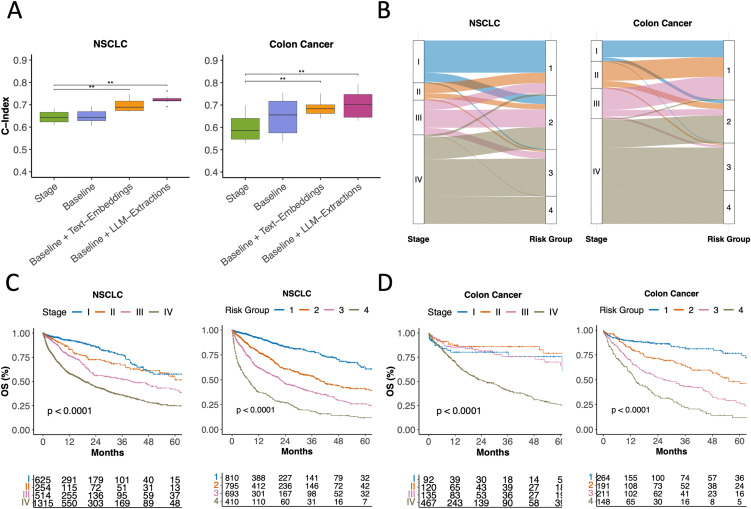
Prognostic Impact of baseline versus LLM-extracted features in NSCLC and colon cancer. **A:** Random Survival Forest results on a 10-fold CV on text embedding and LLM-derived information compared to a baseline model and tumor staging alone. All pairwise comparisons use the paired Wilcoxon signed-rank test. **B:** Sankey diagrams of patient re-stratification from clinical stage to RSF-predicted risk group in NSCLC and colon cancer, using an RSF trained on the combined baseline data and LLM-extracted features. **C:** Kaplan-Meier curves for NSCLC stratified by stage (left) and RSF-generated risk groups (right). **D:** Kaplan-Meier curves for colon cancer stratified by stage (left) and RSF-generated risk groups (right).

Next, we investigated whether unstructured EHR notes could improve survival prediction beyond tumor staging. The text information was used in two different forms: (1) structured features derived by LLM and (2) raw text embeddings generated by a specialized embedding model [15,16]. We trained Random Survival Forest (RSF) models and compared four results: Tumor staging alone, RSF trained on baseline clinical variables (stage, age, sex), RSF trained on baseline plus text embeddings, and RSF trained on baseline plus LLM-derived features [17,18]. Performance was evaluated using ten-fold cross-validated C-indices, with statistical significance determined by a paired Wilcoxon signed-rank test. In the NSCLC cohort, the text-embedding model significantly outperformed TNM staging (0.69 vs. 0.64, p = 0.002, Fig 4A) and the baseline model (0.69 vs. 0.64, p = 0.002). The LLM-extractions model further improved median C-Index significantly over staging alone (0.72 vs. 0.64, p = 0.002), versus the baseline model (0.72 vs 0.64, p = 0.002) and versus the text-embedding model (0.72 vs 0.69, p = 0.014).

These results could be validated in the colon cancer cohort, where the text-embedding model reached a median C-Index of 0.68, outperforming stage (0.59, p = 0.002) and baseline model (0.66, p = 0.193). The LLM-derived model further improved prediction results compared to text embeddings (0.70 vs 0.68, p = 0.275).

To assess model calibration, we compared predicted versus Kaplan-Meier-observed survival probabilities across equidistant bins at 1, 3, and 5 years (S10 Fig). We further evaluated model performance using the Integrated Brier Score (IBS), computed at 1, 3, and 5 years using predicted survival probabilities and accounting for censoring via inverse probability of censoring weighting (IPCW) estimated from the training fold (S10 Fig). The LLM augmented model achieved the lowest mean IBS in both cohorts (NSCLC: 0.206; colon cancer: 0.188), outperforming the baseline and text-embedding models, consistent with the improvements observed in discriminative performance.

### LLM-enhanced prediction model enables improved patient stratification

Using predictions from the RSF model trained on the baseline data combined with the LLM-derived information, we stratified patients into four distinct risk groups according to their predicted cumulative hazards (see Methods, Fig 4B). Comparing the documented tumor stages and the risk groups predicted by our model, we observed that in early-stage patients (I-II), our model reclassified patients only modestly into adjacent risk groups. In contrast, patients with more advanced stages (III-IV) were distributed across all four risk groups. This pattern was largely similar in NSCLC and colon cancer patients.

In Kaplan-Meier analysis, survival curves for both NSCLC and colon cancer cohorts showed more distinct separation between risk groups defined by our machine learning model with LLM-extracted features than between TNM stages (log-rank p < 0.001; Fig 4C and 4D). This enhanced stratification was particularly pronounced among early-stage patients in both cohorts. The greatest absolute impact of the LLM-enhanced model, however, was observed in stage IV, which represented the largest subgroup in both cohorts. Within this group, the model identified substantial prognostic heterogeneity, reclassifying the majority of patients into lower risk groups (NSCLC: 915 out of 1315, 69.6%, colon cancer: 319 out of 467, 68.3%). These findings underscore the potential of LLM-derived unstructured information to uncover prognostic heterogeneity not captured by UICC staging alone. To assess whether model discrimination varied systematically across patient subpopulations, we computed stratified C-indices across age, sex, stage, histology, and available document sources. Discrimination was stable across all subgroups in both cohorts, with no significant pairwise differences after FDR correction (S11 Fig).

### LLM-derived features influence model predictions

**Fig 5:**
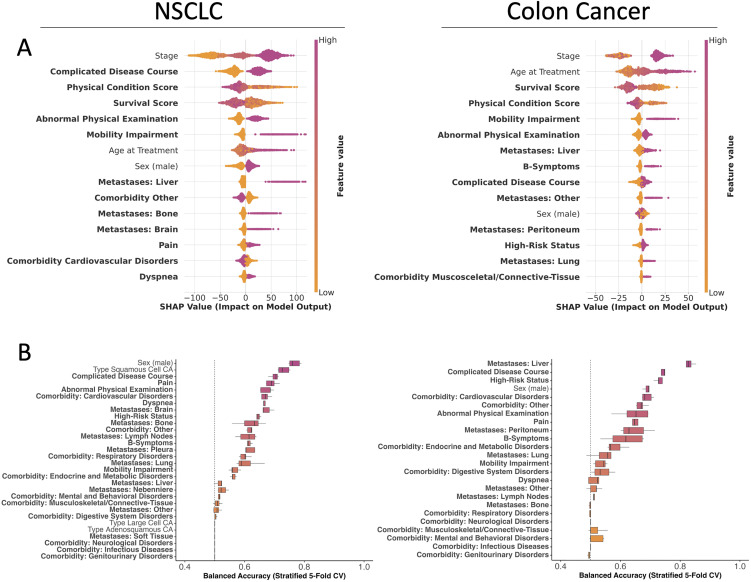
Interpreting model behavior and embedding informativeness. **A:** SHAP summary plots for the top 15 predictors in a Random Survival Forest trained on baseline clinical data plus LLM-extracted features. Features are ordered by their mean absolute SHAP values, quantifying their relative contribution to the predicted survival risk. SHAP distributions are shown over 10 cross-validation test folds. The x-axis represents risk contribution (positive = higher risk), with color indicating feature value (orange = low, purple = high). **B:** Balanced accuracy (stratified 5-fold CV) of logistic regression models trained on text embeddings from EHR notes and discharge reports to predict clinical features (LLM-extracted variables in bold). The dashed vertical line indicates chance‐level balanced accuracy (0.5).

To understand the decision-making process of the LLM-enriched prediction model, we conducted a feature importance analysis using Shapley Additive Explanations (SHAP, Fig 5A) [19]. In both of our cancer cohorts, tumor stage was identified as the most influential variable: Among LLM-derived features, Complicated Disease Course ranked second in NSCLC. The LLM-inferred Survival and Physical Condition scores (third and fourth in importance). Abnormal physical examination, mobility impairment, and older age further increased risk. As expected, metastatic involvement of the liver, bone, or brain also increased predicted risk. The colon cancer model generally showed a similar order of feature importance (Spearman’s ρ = 0.758, p < 0.001). After stage and age, the LLM-derived survival and physical-condition scores had the highest impact on model predictions, followed by other LLM-derived PCIs. Notably, mobility impairment, abnormal examination findings, and B-symptoms outweighed metastatic sites in predictive importance.

### Text embeddings encode patient characteristics

Given the robust performance of the embedding-enhanced prediction model, we investigated the information captured within the text embeddings. As the embedded features are not directly interpretable, we used a surrogate approach using regression models based on text embeddings to predict structured, understandable features (Fig 5B).

In the NSCLC cohort, sex was predicted most accurately (median balanced accuracy = 0.76), followed by squamous cell carcinoma (0.73). Among LLM-derived features, complicated disease course ranked third overall (0.7), followed by pain (0.69), abnormal physical examination (0.69), cardiovascular disorders (0.67), and dyspnea (0.66). All PCIs achieved median balanced accuracies above 0.56, indicating that these clinical features are at least partially encoded in the text-embedding model.

In the colon cancer cohort, despite generally wider interquartile ranges, the results were largely consistent with those observed in the NSCLC cohort (Spearman’s ρ = 0.65, p < 0.001). Liver metastases were the most accurately predicted variable in the colon cancer cohort (balanced accuracy = 0.83), followed by complicated disease course (0.75), high-risk status (0.73), and sex (0.69). Notably, dyspnea was less predictable in this cohort (0.52), likely reflecting the lower prevalence outside of lung cancer.

## Discussion

Multimodal real-world data have become increasingly accessible across all domains and have been used in combination with deep learning models to predict outcomes and guide treatment decisions [20,21]. In addition, a significant proportion of information in EHR consists of unstructured text that remains largely unused by current machine learning approaches [2,22,23]. In this study, we investigated the potential of LLMs for the prediction of real-world patient outcomes based on a large dataset containing pre-treatment EHR notes and discharge summaries from 2,708 patients with NSCLC and a validation cohort of 814 patients with colon cancer.

We implemented two strategies to leverage clinical texts for patient survival prediction. First, we used an LLM for zero-shot information extraction. This resulted in seven patient condition indicators, along with each patient’s comorbidities and sites of metastasis. Comparing the LLM-extracted features with manually curated features showed high similarity, aligning with previous studies, showing the potential of LLM for accurate information extraction [24–26]. To investigate the LLM’s intrinsic medical reasoning capabilities in a minimally guided setting, we further prompted it to generate two composite scores at the patient level: an overall physical status score and a survival score. Notably, we found that both scores have high prognostic value in our real-world cohorts, showing the remarkable medical zero-shot capabilities of current LLM without task-specific training. These results align with recent studies demonstrating that LLMs can infer clinical risk based on patient context, suggesting their potential for general-purpose clinical reasoning [27–29]. Second, we used a text embedding model to transform raw text into high-dimensional feature vectors, a technique previously shown to improve patient outcome prediction [15,30,31].

Comparing models trained on LLM-derived or embedding data, we found that in both approaches, incorporating information from unstructured clinical text significantly improved survival prediction compared to UICC staging alone, with an LLM-informed model achieving the highest performance overall. This enabled the reclassification of a significant proportion of NSCLC and colon cancer patients into four distinct LLM-informed risk groups. By investigating the decision-making of our model, we found that while TNM staging remained the most important feature in both NSCLC and colon cancer cohorts, the two LLM-derived composite scores consistently ranked among the top four most important features. This suggests that the LLM-derived host-level characteristics play a modulatory role, contextualizing tumor-specific information. These findings align with growing evidence that integrating host-specific factors alongside tumor-specific characteristics is critical for optimizing personalized treatment approaches in cancer care [32–35]. Although the black-box nature of text embeddings limits interpretability, our analyses indicate that they also captured host-specific information, potentially contributing to the robust performance of embedding-enhanced survival models in our study.

Our study has limitations, most of which are inherent to the nature of retrospective real-world studies. While inclusion criteria were kept minimal to maintain a representative cohort, selection bias cannot be entirely excluded, as a small proportion of patients with unavailable data were excluded from the analysis. A related challenge is the heterogeneity of oncology documentation, including variability in note frequency and detail, which may introduce biases and distribution shifts. To capture a representative patient population of a large academic cancer center, we included all patients at the time of their first treatment at our institution. However, some patients may have received previous external treatments, which could have influenced LLM decision-making. While this adds variability, it also reflects the realities of routine clinical care. Another limitation is inherent biases from the LLM itself, reflecting possible inaccuracies or outdated information from training datasets, such as obsolete ICD definitions. Furthermore, any inaccuracies in LLM-extracted features may propagate downstream into the survival prediction model, potentially affecting both the estimated risk scores and the resulting patient stratification. While our validation against structured EHR data demonstrated overall high extraction quality, residual extraction errors cannot be fully excluded and may introduce noise which affects the true prognostic signal of individual features. We evaluated the reliability of LLM-based information extraction against expert annotations and structured EHR data of our institution, finding consistent extraction performance across patient subgroups. However, its reliability in diverse hospital systems and international healthcare settings remains an important area of ongoing research.

Our results show the potential of unstructured text to significantly enhance survival prediction and treatment guidance. While embedding models allow straightforward use of unstructured text, LLMs can extract interpretable information at scale, transforming fragmented narratives into actionable features that further enhance prediction models.

Recent advances in open-source and distilled LLMs make it increasingly practical to embed these tools directly into clinical workflows. Practically, the framework presented here could be integrated into existing hospital information systems to automatically flag high-risk patients at the point of treatment planning, enabling earlier escalation of care or inclusion in clinical trials. Beyond outcome prediction, the structured extraction of patient condition indicators from routine notes could reduce documentation burden and support standardized, comparable patient characterization across institutions.

Further research should investigate the clinical utility of report-derived features, specifically exploring how their integration into hospital decision-support systems could automate the identification of high-risk patients for treatment escalation or clinical trial recruitment.

## Methods

### Study design

We retrospectively included 2,708 non-small cell lung cancer (NSCLC) patients and 814 colon cancer patients treated at University Hospital Essen in this study. Overall survival (OS) was defined as the time from initiation of treatment at our institution to death from any cause. The date of death was obtained from either the medical record or, if unavailable, from the state cancer registry. Patients without a documented date of death were right censored on the date of their last clinical visit. The study was approved by the Ethics Committee of the Medical Faculty of the University Duisburg-Essen (No. 22–10881-BO).

### Data acquisition

All medical data used in this study were collected using the Smart Hospital Information Platform (SHIP) of the University Hospital Essen, comprising clinical documents recorded between 2010 and 2025. With SHIP, the data of multiple clinical subsystems are integrated and stored in Fast Healthcare Interoperability Resources (FHIR) format, allowing for data collection based on specific queries. After the collection of NSCLC and colon cancer patients based on available tumor documentation, structured baseline characteristics including age, sex, and tumor stage were directly extracted from the structured EHR fields, while comorbidities were represented by ICD-10 codes automatically extracted from the unstructured clinical notes by the LLM, as described in the Text Processing section below. For the NSCLC cohort, inclusion was restricted to patients with treatment initiation in 2017 or later to ensure a more homogeneous cohort. Patients with missing baseline variables, including age, sex, or tumor stage, were excluded (NSCLC: n = 139; colon cancer: n = 66). Subsequently, patients without any available text report prior to treatment initiation and patients with failed LLM feature extraction were excluded. The full inclusion and exclusion flow is detailed in S1 Fig.

### Text processing and model deployment

We used two approaches to incorporate information from unstructured clinical text into our analyses:

1. **LLM Feature Extraction:** We used Meta’s multimodal large language model, Llama 4 Scout, to extract pre-specified features in this study based on a uniform prompt in a zero-shot manner (S12 Fig). The Scout model, a version of the Llama 4 series, features 17 billion active parameters, with a total of 109 billion parameters distributed across 16 experts. Llama 4 Scout has been deployed locally using Hugging Face’s Text Generation Interference (TGI) on four NVIDIA H100 GPUs, operating with a context window of 8,000 tokens. The prompt engineering strategy employed a persona-based approach, directing the model to act as a clinical expert in oncology and utilize logical deduction to extract or infer features from the clinical text. In particular, we extracted metastasis locations and comorbidities as lists, representing comorbidities by ICD codes. Furthermore, we encoded the presence or absence of B-symptoms, dyspnea, pain, high-risk status, complicated disease course, abnormal physical examination, and mobility impairment as binary features. Beyond these, we prompted the LLM to generate two composite scores based on the reports: a Physical Condition Score (ranging from 0 to 100, where scores closer to 0 indicate worse physical condition) and a Survival Score (ranging from 0 to 100, where 100 indicates a higher likelihood of survival). To ensure technical reproducibility and downstream compatibility, the prompt specified a valid JSON output format without additional conversational text. Outputs were given in JSON format, and cases with failed extraction were excluded from subsequent analyses (S1 Fig).
2. **Text embeddings:** To generate high-dimensional vector representations directly from the raw clinical text, we used the multi-lingual BGE-M3 model, which is capable of handling inputs of varying sizes [15]. A maximal chunk size of 512 tokens and a sliding window size of 32 tokens was used for dense retrieval.

To ensure the confidentiality of patient data, the text reports were exclusively processed within the hospital network. All models were internally deployed using the KI Translation Essen (KITE) platform. KITE, a research infrastructure of the University Hospital Essen and the Medical Faculty of the University of Duisburg-Essen, supports the translation of algorithms to the point of care. Furthermore, this study adheres to the TRIPOD-LLM reporting guidelines for studies developing or validating prediction models using large language models, and the completed checklist has been provided as supplementary information (S1 Checklist) [36].

### Datasets

For evaluation, we created three datasets to assess whether these two approaches for information extraction/embedding can improve survival prediction as measured by the C-Index. Therefore, we created:

1. **Stage Dataset:**

This dataset includes only the tumor stage information (I-IV) of each patient.

2. **Baseline Dataset:**

The stage dataset was expanded through the incorporation of additional patient demographics and clinical variables, including sex, age, and, for patients with NSCLC, tumor type. This expanded dataset was designated as the baseline dataset. We then enriched the baseline dataset with features extracted and embeddings generated by an LLM. To maximize patient inclusion, we merged data from both EHR notes and discharge reports, retaining patients with at least one available report prior to treatment initiation. When both report types were available, binary and categorical features were merged using a logical OR operation, for example, a feature absent in the EHR note but present in the discharge report was set to true. Comorbidities and metastases were dummy-encoded prior to merging. For continuous features, including LLM-derived composite scores, the element-wise mean across both reports was computed. For text embeddings specifically, the element-wise mean of the two 1,024-dimensional vectors was computed before applying PCA for dimensionality reduction, retaining the top 30 principal components. When only a single report type was available, its features and embeddings were used directly without aggregation. This merging procedure was applied identically across all model variants, yielding a single harmonized patient subset on which all models, staging alone, baseline, baseline with text embeddings, and baseline with LLM-extracted features, were subsequently trained and evaluated.

### Curating LLM-extracted features

We identified common comorbidities using ICD codes and grouped them into clinically relevant categories. To focus on non-cancer comorbidities, we excluded ICD codes that start with “C”. We identified risk groups by ICD code chapter, Cardiovascular Disorders (I), Respiratory Disorders (J), Endocrine and Metabolic Disorders (E), Infectious Diseases (A, B, U), Neurological Disorders (G), Digestive System Disorders (K), Genitourinary Disorders (N), Musculoskeletal and Connective Tissue Disorders (M), and Mental and Behavioral Disorders (F), and assigned all remaining codes to an Other category. For metastases, we standardized the extracted terms, accounting for variations like plural versus singular forms and abbreviations, by mapping them to common metastases locations. We retained only those metastases occurring in at least 25 reports, applying the same grouping strategy as with ICD codes, where unmatched terms were assigned to “Other”. Absence of comorbidities was encoded by setting all comorbidity risk group flags to False, while absence of metastases was encoded separately by setting all metastasis flags to False.

### Survival model

We trained a Random Survival Forest (RSF) with 1,000 trees, a minimum of five samples required to split an internal node, and at least five samples per leaf, using 10-fold cross-validation on the three datasets, Baseline, Baseline + LLM-extractions and Baseline + text-embeddings. To derive a five-year risk score, we set a reference time T_ref of 1825 days and evaluated each test patient’s cumulative hazard function at T_ref. That hazard value served as the patient’s risk score, which we then partitioned into four groups. To limit the influence of extreme values, we excluded scores below the 2.5th percentile and above the 97.25th percentile when calculating bin boundaries, performed equal-width binning on the remaining range, and assigned all excluded scores to the lowest or highest bin as appropriate.

### Statistics

Prior to model training, the aggregated 1,024-dimensional text embedding vectors, computed as described above, were reduced to 30 principal components via PCA using *scikit-learn’s* default settings [37]. Continuous features were standardized using *scikit-learn’s* StandardScaler, and categorical features were one-hot encoded with its MultiLabelBinarizer. Kaplan-Meier curves were estimated and compared by the log-rank test, and Cox proportional hazards models were fitted using the *survival* package in R [38,39]. Random Survival Forests were trained independently on each dataset using tenfold cross-validation in Python’s *scikit-survival* package to estimate C-Indices, which were then compared across datasets by paired Wilcoxon signed-rank test in R’s *stats* package [40]. SHAP values were calculated with the model-agnostic explainer from the Python *shap* library for each fold and aggregated into a single explainer object, enabling consistent, global interpretation of feature contributions across the entire dataset [19]. Spearman correlations of SHAP values were computed using Python’s *scipy* package, and Pearson correlations between LLM-inferred scores and patient characteristics were calculated with R’s *stats* package [41].

### Visualizations

Fig 1 was created with BioRender.com.

## Supporting information

S1 Checklist TRIPOD-LLM checklist. (PDF)

S1 Fig Flow diagram for patients included in the analyses. (DOCX)

S2 Fig Comparison of metastatic site frequencies in stage IV NSCLC and colon cancer between a structured clinical database and LLM-based extraction. (DOCX)

S3 Fig Validation of LLM-based comorbidity extraction. (DOCX)

S4 Fig Correlation between clinical text length and LLM-inferred scores. (DOCX)

S5 Fig Histogram distributions of LLM-created composite scores for physical condition and survival in NSCLC and colon cancer cohorts. (DOCX)

S6 Fig Association of LLM-inferred scores with patient characteristics. (DOCX)

S7 Fig Validation of LLM-derived physical condition score against structured ECOG performance status. (DOCX)

S8 Fig Excerpts from clinical notes of four patients and their corresponding LLM-based interpretations. (DOCX)

S9 Fig Kaplan-Meier survival curves for colon cancer patients stratified by LLM-inferred physical condition and survival scores at disease stages I–IV. (DOCX)

S10 Fig Integrated Brier Score and calibration of survival models in NSCLC and colon cancer. (DOCX)

S11 Fig Subgroup analysis of model discrimination in NSCLC and colon cancer cohorts. (DOCX)

S12 Fig Base prompt for LLM-driven information retrieval from clinical notes. (DOCX)

S1 Table Validation of LLM-extracted patient condition indicators in the NSCLC cohort. (DOCX)

S2 Table Validation of LLM-extracted patient condition indicators in the colon cancer cohort. (DOCX)

S3 Table LLM extraction accuracy by patient subgroup, NSCLC cohort. (DOCX)

S4 Table LLM extraction accuracy by patient subgroup, colon cancer cohort. (DOCX)

S5 Table Results of univariate Cox proportional-hazards models evaluating the association between LLM-extracted covariates and overall survival. (DOCX)

S6 Table Results of univariate and multivariate Cox proportional-hazards models for the NSCLC cohort evaluating the association between LLM-inferred scores and overall survival. (DOCX)

S7 Table Results of univariate and multivariate Cox proportional-hazards models for the colon cancer cohort evaluating the association between LLM-inferred scores and overall survival. (DOCX)

## References

[1] Chen RJ, Lu MY, Williamson DFK, Chen TY, Lipkova J, Noor Z, et al. Pan-cancer integrative histology-genomic analysis via multimodal deep learning. Cancer Cell. 2022;40(8):865-878.e6. doi: 10.1016/j.ccell.2022.07.004 35944502 PMC10397370

[2] Keyl J, Keyl P, Montavon G, Hosch R, Brehmer A, Mochmann L, et al. Decoding pan-cancer treatment outcomes using multimodal real-world data and explainable artificial intelligence. Nat Cancer. 2025;6(2):307–22. doi: 10.1038/s43018-024-00891-1 39885364 PMC11864985

[3] Keyl J, Kasper S, Wiesweg M, Götze J, Schönrock M, Sinn M, et al. Multimodal survival prediction in advanced pancreatic cancer using machine learning. ESMO Open. 2022;7(5):100555. doi: 10.1016/j.esmoop.2022.100555 35988455 PMC9588888

[4] Schulz S, Woerl A-C, Jungmann F, Glasner C, Stenzel P, Strobl S, et al. Multimodal deep learning for prognosis prediction in renal cancer. Front Oncol. 2021;11:788740. doi: 10.3389/fonc.2021.788740 34900744 PMC8651560

[5] Steyaert S, Qiu YL, Zheng Y, Mukherjee P, Vogel H, Gevaert O. Multimodal deep learning to predict prognosis in adult and pediatric brain tumors. Commun Med (Lond). 2023;3(1):44. doi: 10.1038/s43856-023-00276-y 36991216 PMC10060397

[6] Steyaert S, Pizurica M, Nagaraj D, Khandelwal P, Hernandez-Boussard T, Gentles AJ, et al. Multimodal data fusion for cancer biomarker discovery with deep learning. Nat Mach Intell. 2023;5(4):351–62. doi: 10.1038/s42256-023-00633-5 37693852 PMC10484010

[7] Wu X, Zhang S, Zhang Z, He Z, Xu Z, Wang W, et al. Biologically interpretable multi-task deep learning pipeline predicts molecular alterations, grade, and prognosis in glioma patients. NPJ Precis Oncol. 2024;8(1):181. doi: 10.1038/s41698-024-00670-2 39152182 PMC11329669

[8] Wong C, et al. Universal abstraction: harnessing frontier models to structure real-world data at scale. Preprint; 2025. Available from: doi: 10.48550/arXiv.2502.00943

[9] Wiest IC, Wolf F, Leßmann M-E, van Treeck M, Ferber D, Zhu J, et al. LLM-AIx: an open source pipeline for Information Extraction from unstructured medical text based on privacy preserving Large Language Models. medRxiv. 2024:2024.09.02.24312917. doi: 10.1101/2024.09.02.24312917 39281753 PMC11398444

[10] Wiest IC, Ferber D, Zhu J, van Treeck M, Meyer SK, Juglan R, et al. Privacy-preserving large language models for structured medical information retrieval. NPJ Digit Med. 2024;7(1):257. doi: 10.1038/s41746-024-01233-2 39304709 PMC11415382

[11] Touvron H, et al. LLaMA: open and efficient foundation language models. Preprint; 2023. Available from: doi: 10.48550/arXiv.2302.13971

[12] Team G, et al. Gemma: open models based on gemini research and technology. Preprint; 2024. Available from: doi: 10.48550/arXiv.2403.08295

[13] Wornow M, Xu Y, Thapa R, Patel B, Steinberg E, Fleming S, et al. The shaky foundations of large language models and foundation models for electronic health records. NPJ Digit Med. 2023;6(1):135. doi: 10.1038/s41746-023-00879-8 37516790 PMC10387101

[14] Markowetz F. All models are wrong and yours are useless: making clinical prediction models impactful for patients. NPJ Precis Oncol. 2024;8(1):54. doi: 10.1038/s41698-024-00553-6 38418530 PMC10901807

[15] Chen J, et al. BGE M3-embedding: multi-lingual, multi-functionality, multi-granularity text embeddings through self-knowledge distillation. Preprint; 2024. Available from: doi: 10.48550/arXiv.2402.03216

[16] The Llama 4 herd: the beginning of a new era of natively multimodal AI innovation. Meta AI. Available from: https://ai.meta.com/blog/llama-4-multimodal-intelligence/

[17] Ishwaran H, Kogalur UB, Blackstone EH, Lauer MS. Random survival forests. Ann Appl Stat. 2008;2:841–60.

[18] Ishwaran H, Gerds TA, Kogalur UB, Moore RD, Gange SJ, Lau BM. Random survival forests for competing risks. Biostatistics. 2014;15(4):757–73. doi: 10.1093/biostatistics/kxu010 24728979 PMC4173102

[19] Lundberg S, Lee S-I. A unified approach to interpreting model predictions. Preprint; 2017. Available from: doi: 10.48550/arXiv.1705.07874

[20] Singhal K, Azizi S, Tu T, Mahdavi SS, Wei J, Chung HW, et al. Large language models encode clinical knowledge. Nature. 2023;620(7972):172–80. doi: 10.1038/s41586-023-06291-2 37438534 PMC10396962

[21] Saab K, et al. Capabilities of gemini models in medicine. Preprint; 2024. Available from: doi: 10.48550/arXiv.2404.18416

[22] Jee J, et al. Automated real-world data integration improves cancer outcome prediction. Nat. 2024;636:728–36. 10.1038/s41586-024-08167-5 PMC11655358 39506116

[23] Eisemann N, Bunk S, Mukama T, Baltus H, Elsner SA, Gomille T, et al. Nationwide real-world implementation of AI for cancer detection in population-based mammography screening. Nat Med. 2025;31(3):917–24. doi: 10.1038/s41591-024-03408-6 39775040 PMC11922743

[24] Boyle JS, Kascenas A, Lok P, Liakata M, O’Neil AQ. Automated clinical coding using off-the-shelf large language models. Preprint; 2023. Available from: doi: 10.48550/arXiv.2310.06552

[25] Grothey B, Odenkirchen J, Brkic A, Schömig-Markiefka B, Quaas A, Büttner R, et al. Comprehensive testing of large language models for extraction of structured data in pathology. Commun Med (Lond). 2025;5(1):96. doi: 10.1038/s43856-025-00808-8 40164789 PMC11958830

[26] Reichenpfader D, Müller H, Denecke K. A scoping review of large language model based approaches for information extraction from radiology reports. NPJ Digit Med. 2024;7(1):222. doi: 10.1038/s41746-024-01219-0 39182008 PMC11344824

[27] Han C, Kim DW, Kim S, Chan You S, Park JY, Bae S, et al. Evaluation of GPT-4 for 10-year cardiovascular risk prediction: insights from the UK Biobank and KoGES data. iScience. 2024;27(2):109022. doi: 10.1016/j.isci.2024.109022 38357664 PMC10865411

[28] Kavak EE, Erdat EC, Altundağ Derin Z, Dilli İ, Kubilay Tolunay P, Öksüzoğlu B, et al. Comparison of AI chatbot predicted and realworld survival outcomes in hepatocellular carcinoma. Sci Rep. 2025;15(1):21728. doi: 10.1038/s41598-025-06591-9 40596129 PMC12219167

[29] Jiang LY, Liu XC, Nejatian NP, Nasir-Moin M, Wang D, Abidin A, et al. Health system-scale language models are all-purpose prediction engines. Nature. 2023;619(7969):357–62. doi: 10.1038/s41586-023-06160-y 37286606 PMC10338337

[30] Amrollahi F, Shashikumar SP, Razmi F, Nemati S. Contextual embeddings from clinical notes improves prediction of sepsis. AMIA Annu Symp Proc. 2021;2020:197–202. 33936391 PMC8075484

[31] Kim S, Lee C-K, Choi Y, Baek ES, Choi JE, Lim JS, et al. Deep-learning-based natural language processing of serial free-text radiological reports for predicting rectal cancer patient survival. Front Oncol. 2021;11:747250. doi: 10.3389/fonc.2021.747250 34868947 PMC8635726

[32] Panigrahi G, Ambs S. How comorbidities shape cancer biology and survival. Trends Cancer. 2021;7(6):488–95. doi: 10.1016/j.trecan.2020.12.010 33446449 PMC8137526

[33] The importance of aging in cancer research. Nat Aging. 2022;2(5):365–6. doi: 10.1038/s43587-022-00231-x 37118075

[34] Keyl J, Bucher A, Jungmann F, Hosch R, Ziller A, Armbruster R, et al. Prognostic value of deep learning-derived body composition in advanced pancreatic cancer-a retrospective multicenter study. ESMO Open. 2024;9(1):102219. doi: 10.1016/j.esmoop.2023.102219 38194881 PMC10837775

[35] Keyl J, Hosch R, Berger A, Ester O, Greiner T, Bogner S, et al. Deep learning-based assessment of body composition and liver tumour burden for survival modelling in advanced colorectal cancer. J Cachexia Sarcopenia Muscle. 2023;14(1):545–52. doi: 10.1002/jcsm.13158 36544260 PMC9891942

[36] Gallifant J, Afshar M, Ameen S, Aphinyanaphongs Y, Chen S, Cacciamani G, et al. The TRIPOD-LLM reporting guideline for studies using large language models. Nat Med. 2025;31(1):60–9. doi: 10.1038/s41591-024-03425-5 39779929 PMC12104976

[37] Pedregosa F, et al. Scikit-learn: machine learning in Python. J Mach Learn Res. 2011;12:2825–30.

[38] R Core Team. R: a language and environment for statistical computing. Vienna, Austria: R Foundation for Statistical Computing; 2022.

[39] Therneau TM. A package for survival analysis in R; 2023.

[40] Pölsterl S. Scikit-survival: a library for time-to-event analysis built on top of scikit-learn. J Mach Learn Res. 2020;21:1–6.34305477

[41] Virtanen P, Gommers R, Oliphant TE, Haberland M, Reddy T, Cournapeau D, et al. SciPy 1.0: fundamental algorithms for scientific computing in Python. Nat Methods. 2020;17(3):261–72. doi: 10.1038/s41592-019-0686-2 32015543 PMC7056644

