# Supplementary figures and images for: Large language models enable prognostic stratification of cancer patients using real-world clinical notes
Source: PLOS Digit Health. 2026 Jul 8;5(7):e0001546. doi: 10.1371/journal.pdig.0001546 (PMC13345263; doi:10.1371/journal.pdig.0001546)

**
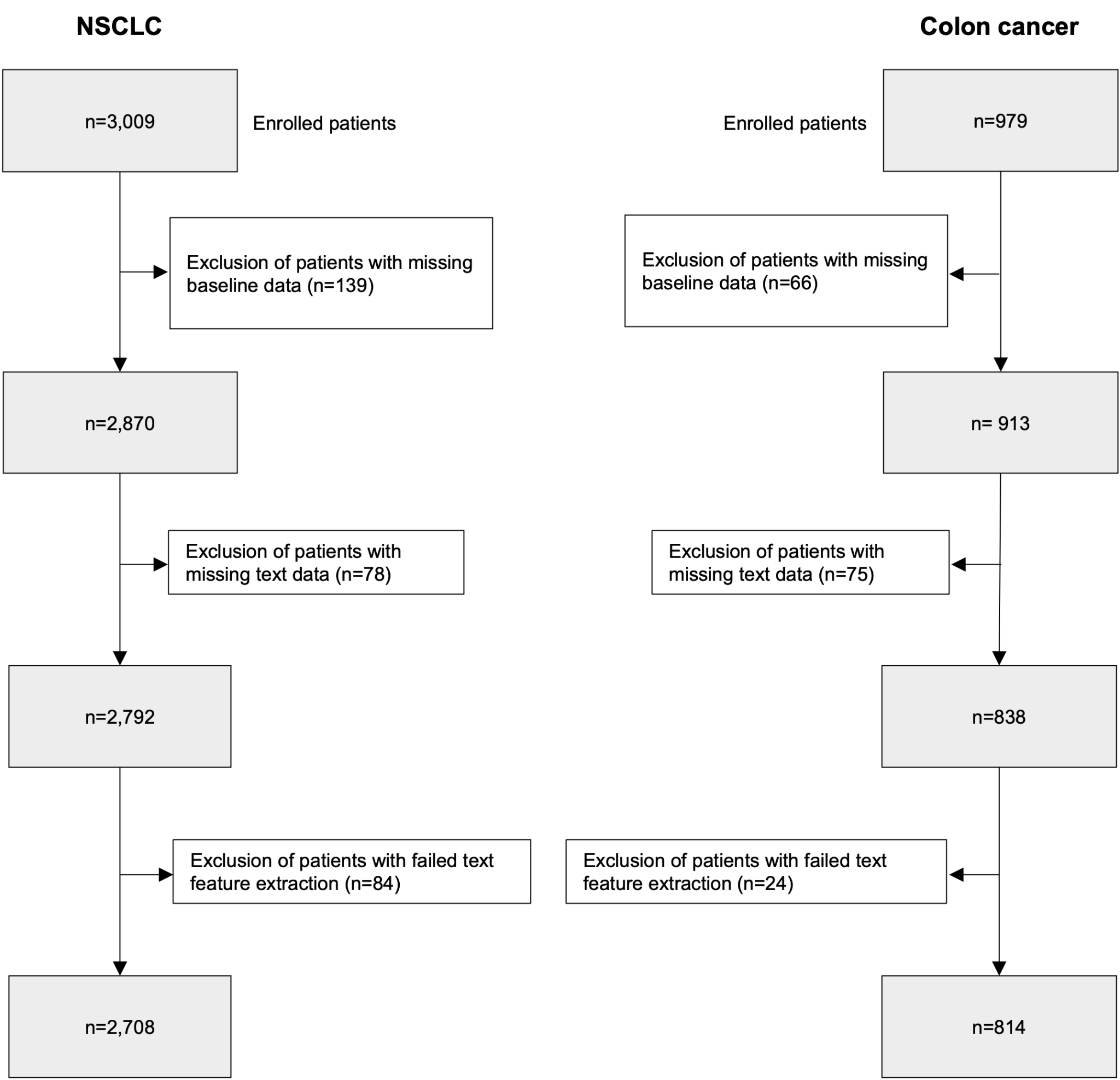
**

**S1 Fig: Flow diagram for patients included in the analyses.**

Supplement: S1 Fig — (DOCX) [file pdig.0001546.s002.docx]

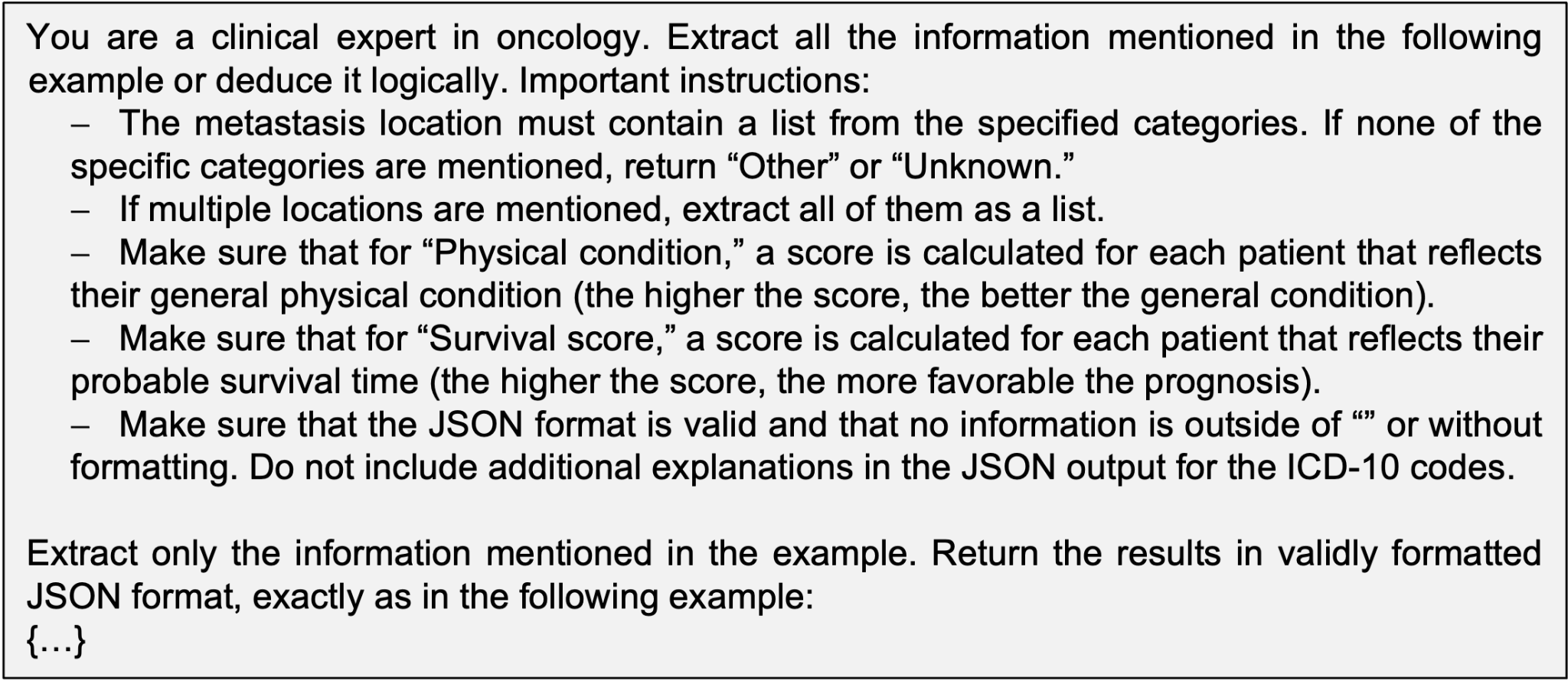


**S12 Fig: Base prompt for LLM-driven information retrieval from clinical notes.**

Supplement: S12 Fig — (DOCX) [file pdig.0001546.s013.docx]
