# Supplementary material for: Large language models enable prognostic stratification of cancer patients using real-world clinical notes
Source: PLOS Digit Health. 2026 Jul 8;5(7):e0001546. doi: 10.1371/journal.pdig.0001546 (PMC13345263; doi:10.1371/journal.pdig.0001546)
Supplement: S2 Fig — (DOCX) [file pdig.0001546.s003.docx]

**
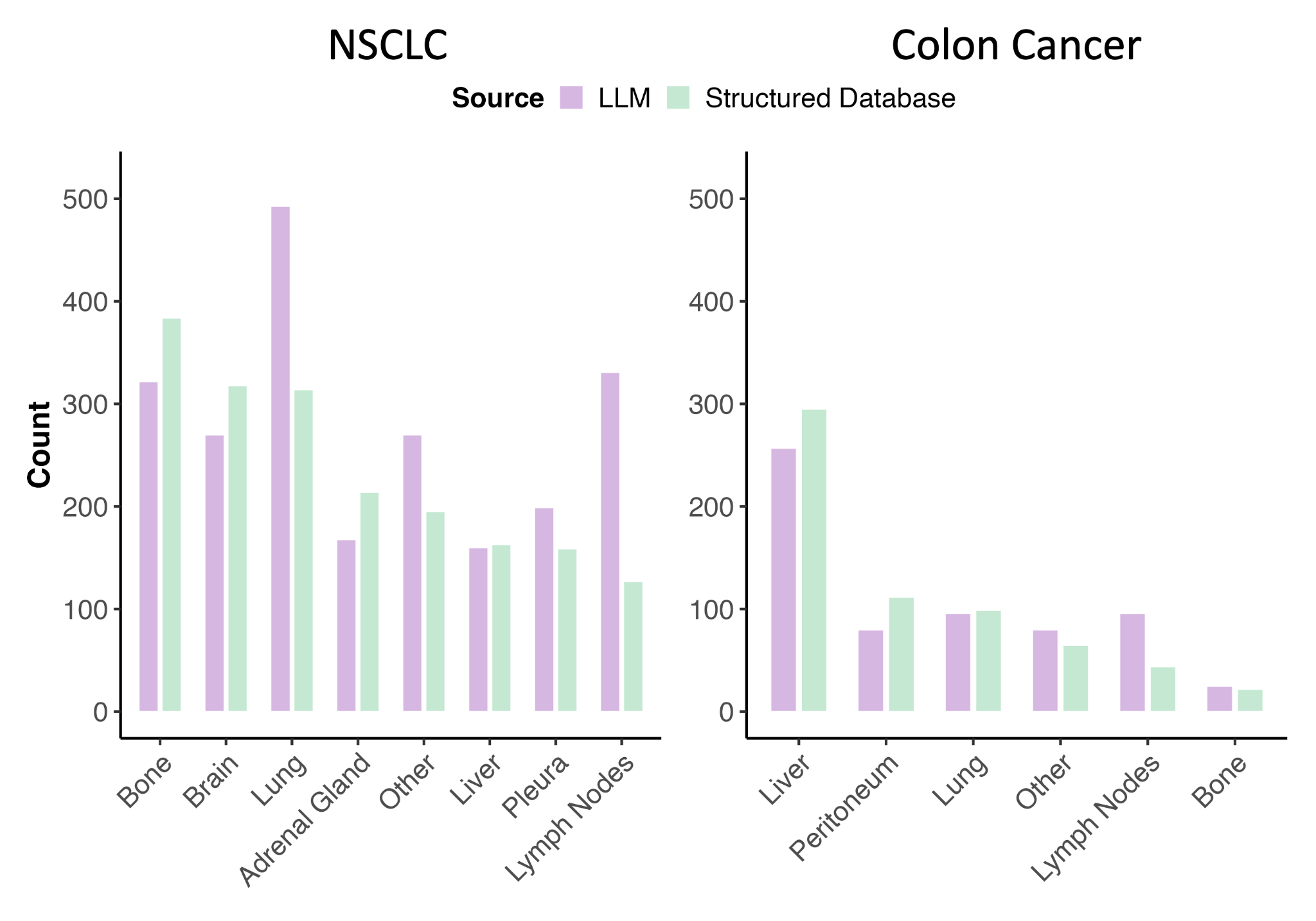
**

**S2 Fig: Comparison of metastatic site frequencies in stage IV NSCLC (left) and colon cancer (right) between a structured clinical database (green) and our LLM‑based extraction from the unstructured medical text records (purple).** Each bar shows the number of patients with metastases at a given anatomical location, sites that together account for less than 3 % of all metastasis occurrences across both sources (threshold = total counts × 0.03) were pooled into the “Other” category.
