# Supplementary material for: Large language models enable prognostic stratification of cancer patients using real-world clinical notes
Source: PLOS Digit Health. 2026 Jul 8;5(7):e0001546. doi: 10.1371/journal.pdig.0001546 (PMC13345263; doi:10.1371/journal.pdig.0001546)
Supplement: S3 Fig — (DOCX) [file pdig.0001546.s004.docx]

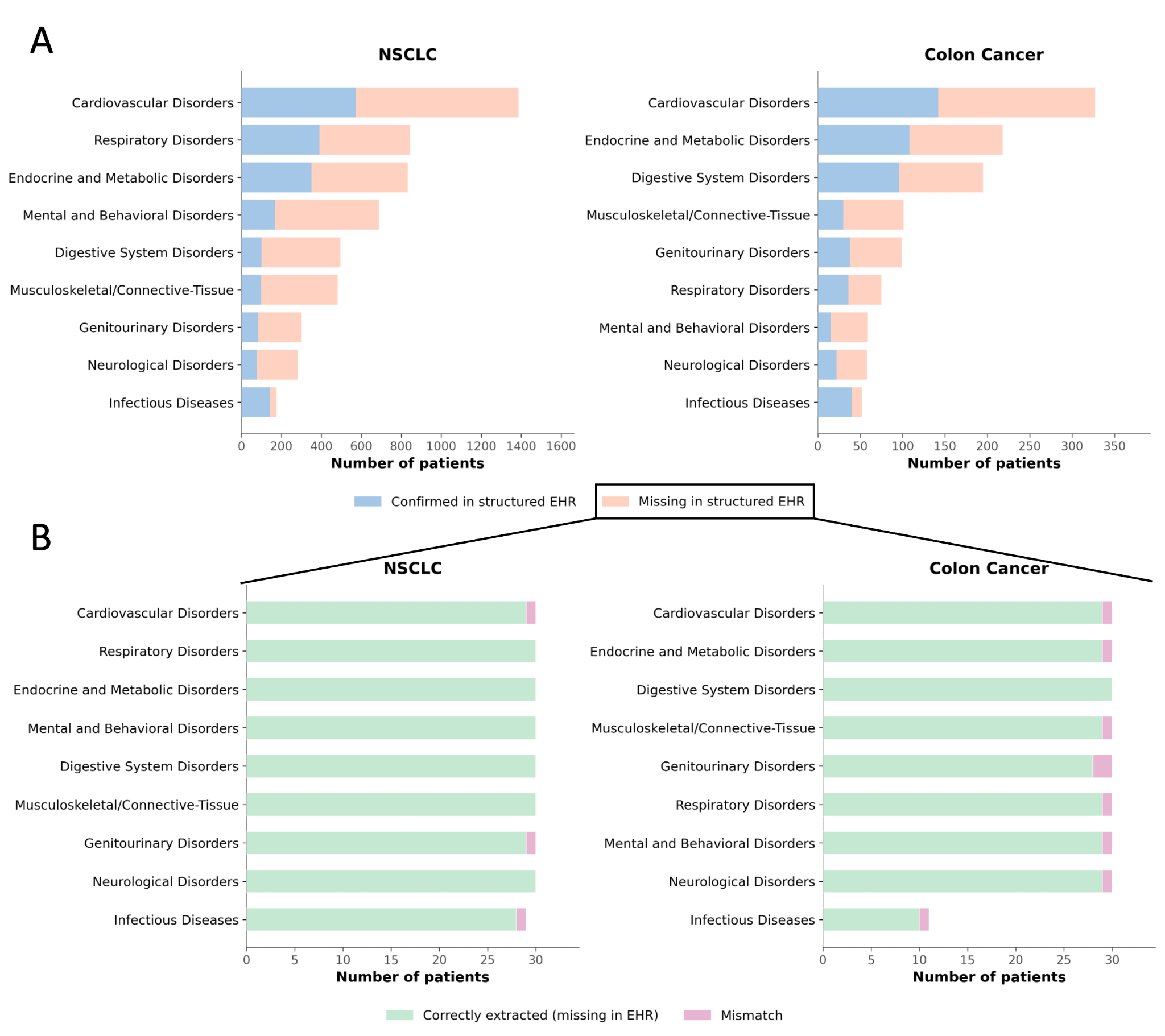


**S3 Fig: Validation of LLM-based comorbidity extraction. A:** Comparison of LLM-extracted comorbidity groups against structured ICD-10 codes from the electronic health record (EHR) in the NSCLC (left) and colon cancer (right) cohorts. Bars show the number of patients with a given comorbidity group extracted by the LLM, stratified by whether the comorbidity was also documented in the structured EHR (blue) or absent from it (orange). **B:** Physician-based manual review of LLM-extracted comorbidities that were missing from the structured EHR. For each comorbidity group, 30 randomly sampled cases were reviewed to distinguish true extractions missing in the structured EHR (green) from false extractions (pink). For infectious diseases, all available cases missing in the structured EHR were annotated.
