# Supplementary material for: Large language models enable prognostic stratification of cancer patients using real-world clinical notes
Source: PLOS Digit Health. 2026 Jul 8;5(7):e0001546. doi: 10.1371/journal.pdig.0001546 (PMC13345263; doi:10.1371/journal.pdig.0001546)
Supplement: S4 Fig — (DOCX) [file pdig.0001546.s005.docx]

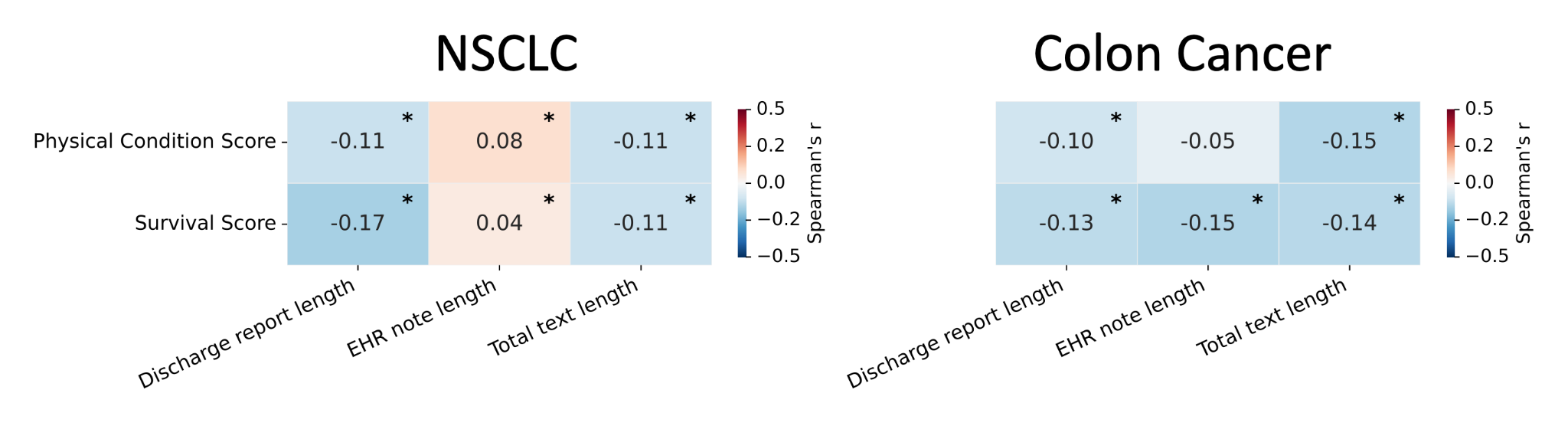


**S4 Fig: Correlation between clinical text length and LLM-inferred scores.** Spearman correlation heatmaps for the NSCLC and colon cancer cohorts. Text length was computed separately for discharge reports, EHR notes, and their combined total, and correlated against the LLM-inferred physical condition and survival scores. Asterisks denote p < 0.05.
