# Supplementary material for: Large language models enable prognostic stratification of cancer patients using real-world clinical notes
Source: PLOS Digit Health. 2026 Jul 8;5(7):e0001546. doi: 10.1371/journal.pdig.0001546 (PMC13345263; doi:10.1371/journal.pdig.0001546)
Supplement: S5 Fig — (DOCX) [file pdig.0001546.s006.docx]

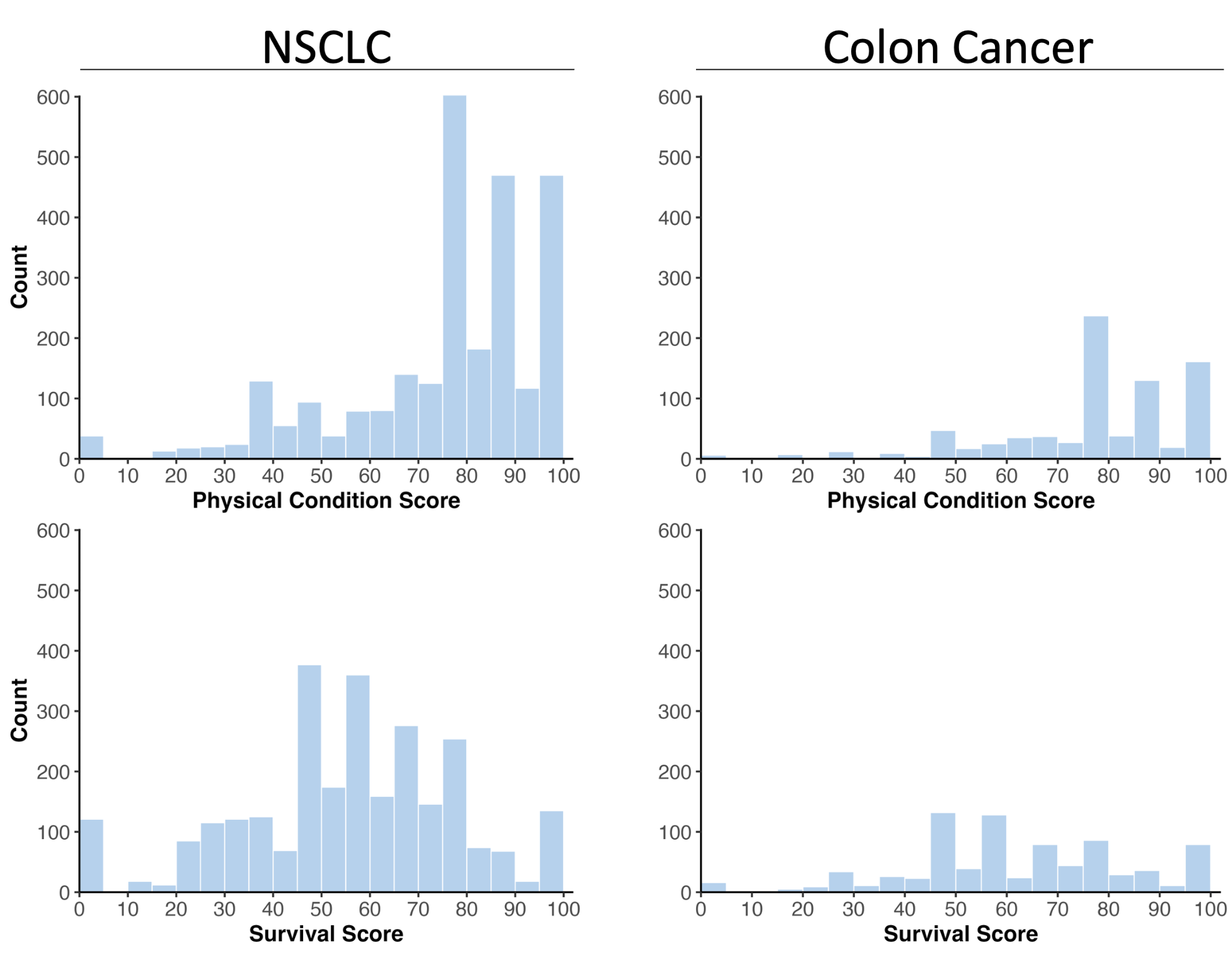


**S5 Fig: Histogram distributions of LLM-created composite scores for physical condition and survival in NSCLC and colon cancer cohorts.**
