# Supplementary material for: Large language models enable prognostic stratification of cancer patients using real-world clinical notes
Source: PLOS Digit Health. 2026 Jul 8;5(7):e0001546. doi: 10.1371/journal.pdig.0001546 (PMC13345263; doi:10.1371/journal.pdig.0001546)
Supplement: S6 Fig — (DOCX) [file pdig.0001546.s007.docx]

**
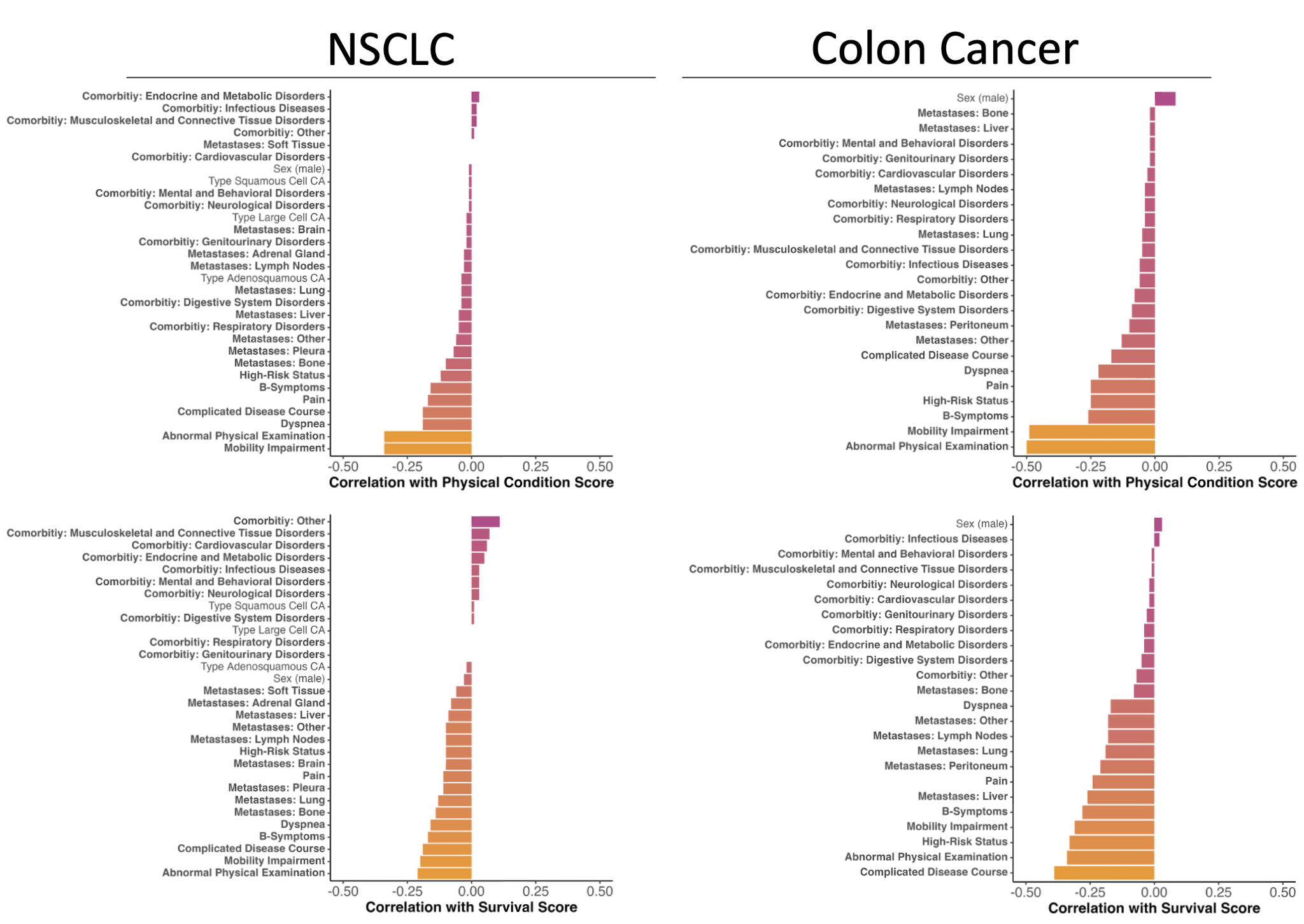
S6 Fig:** **Association of LLM-inferred scores with patient characteristics.** Pearson correlations between each binary feature and the two continuous scores: physical condition (top row) and survival (bottom row) for the NSCLC (left) and colon cancer (right) cohorts. Feature names highlighted in bold were extracted by the LLM.
