# Supplementary material for: Large language models enable prognostic stratification of cancer patients using real-world clinical notes
Source: PLOS Digit Health. 2026 Jul 8;5(7):e0001546. doi: 10.1371/journal.pdig.0001546 (PMC13345263; doi:10.1371/journal.pdig.0001546)
Supplement: S7 Fig — (DOCX) [file pdig.0001546.s008.docx]

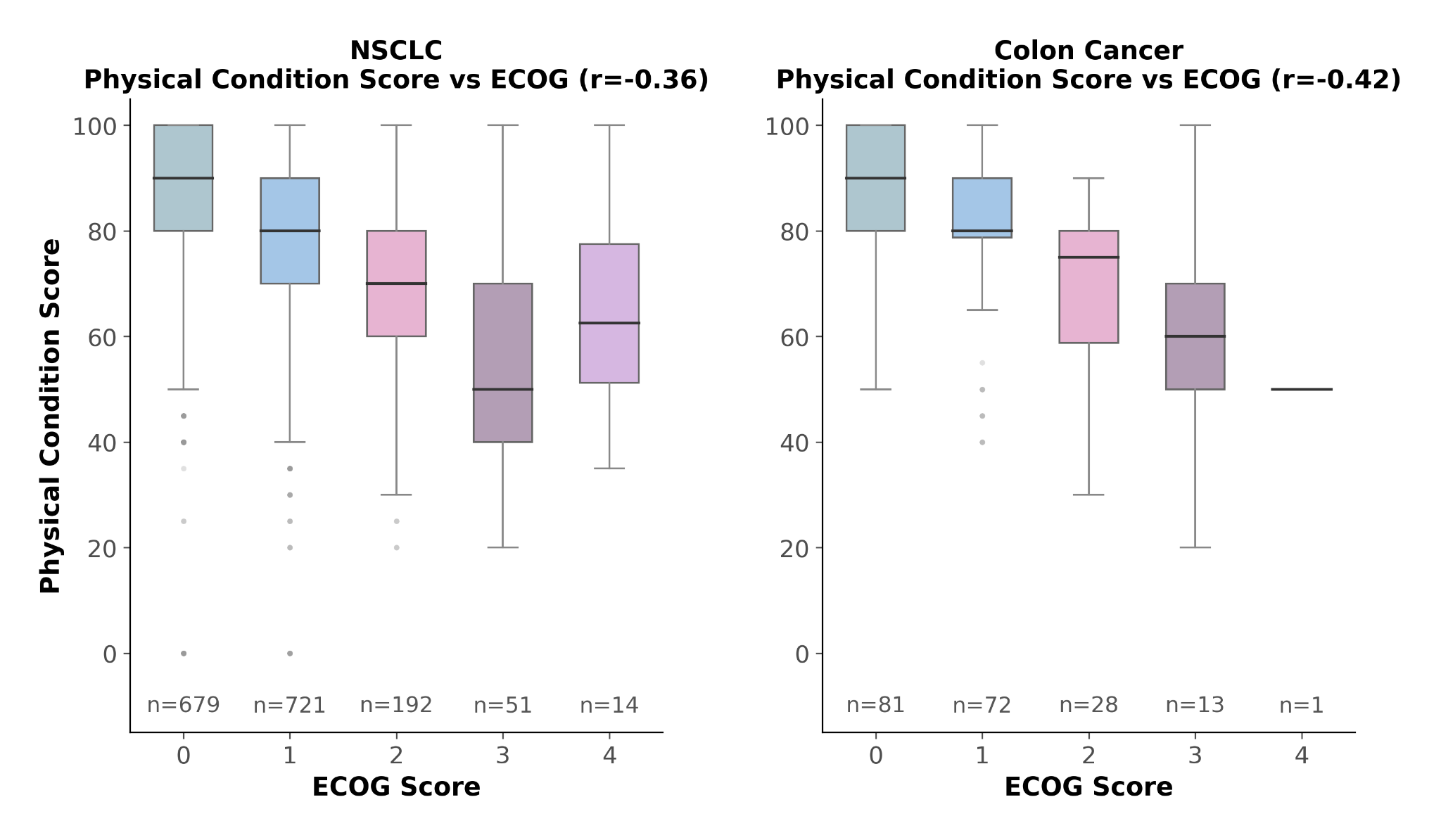


**S7 Fig: Validation of LLM-derived physical condition score against structured ECOG performance status.** Box plots of the LLM-inferred physical condition score stratified by ECOG performance status in the NSCLC (left) and colon cancer (right) cohorts. ECOG scores were available from structured electronic health records for a subset of patients prior to treatment initiation (NSCLC: n=1,657; colon cancer: n=195). Spearman correlation coefficients are indicated in the panel titles (NSCLC: r=−0.36, p<0.001; colon cancer: r=−0.42, p<0.001). Sample sizes per ECOG group are shown below each box.
