# Supplementary material for: Large language models enable prognostic stratification of cancer patients using real-world clinical notes
Source: PLOS Digit Health. 2026 Jul 8;5(7):e0001546. doi: 10.1371/journal.pdig.0001546 (PMC13345263; doi:10.1371/journal.pdig.0001546)
Supplement: S8 Fig — (DOCX) [file pdig.0001546.s009.docx]

**
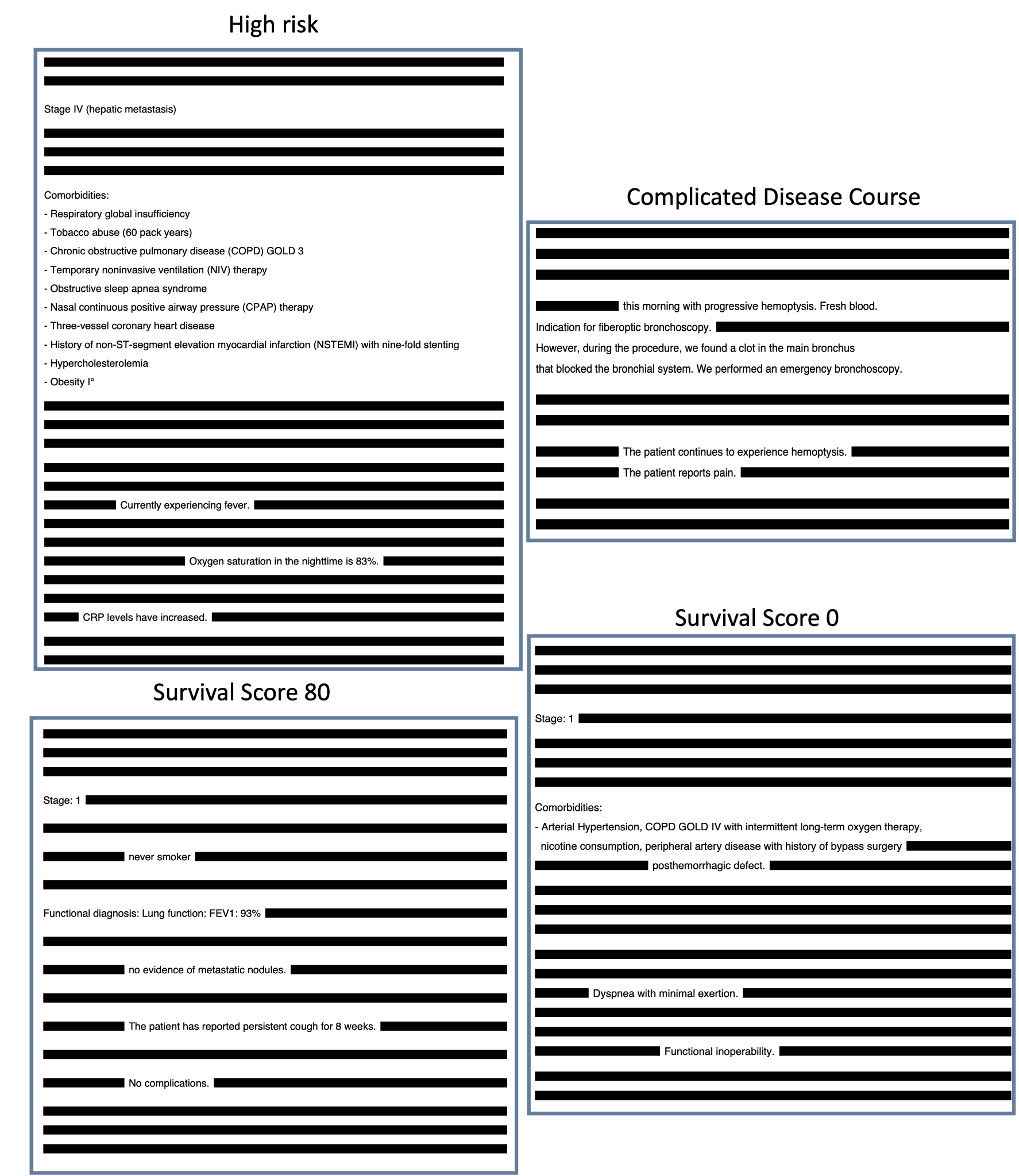
**

**S8 Fig:** Excerpts from clinical notes of four patients and their corresponding LLM-based interpretations, illustrating predictions for a high-risk patient, a complicated disease course, and survival scores of 80 and 0. Text has been translated from German; only selected excerpts are shown for privacy reasons.
