# Supplementary material for: Large language models enable prognostic stratification of cancer patients using real-world clinical notes
Source: PLOS Digit Health. 2026 Jul 8;5(7):e0001546. doi: 10.1371/journal.pdig.0001546 (PMC13345263; doi:10.1371/journal.pdig.0001546)
Supplement: S9 Fig — (DOCX) [file pdig.0001546.s010.docx]

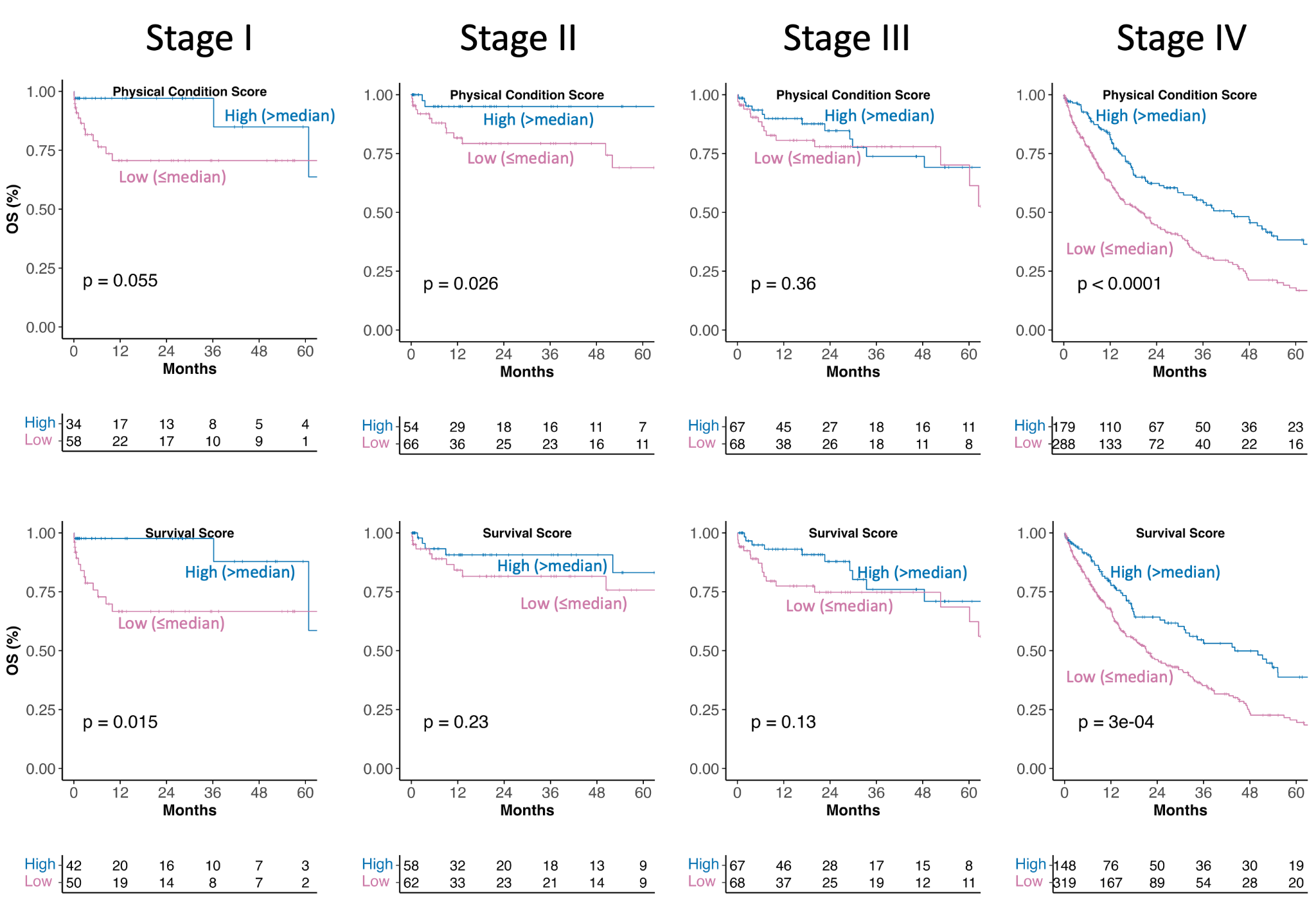


**S9 Fig: Kaplan-Meier survival curves for colon cancer patients stratified by LLM‑inferred physical condition and survival scores at disease stages I–IV.** Patients were dichotomized by the median within their respective stage into high‑score (> median; blue) and low‑score (≤ median; magenta) groups. Survival differences between groups were assessed by the log‑rank test, and corresponding p‑values are indicated in each panel.
