# Supplementary material for: Large language models enable prognostic stratification of cancer patients using real-world clinical notes
Source: PLOS Digit Health. 2026 Jul 8;5(7):e0001546. doi: 10.1371/journal.pdig.0001546 (PMC13345263; doi:10.1371/journal.pdig.0001546)
Supplement: S10 Fig — (DOCX) [file pdig.0001546.s011.docx]

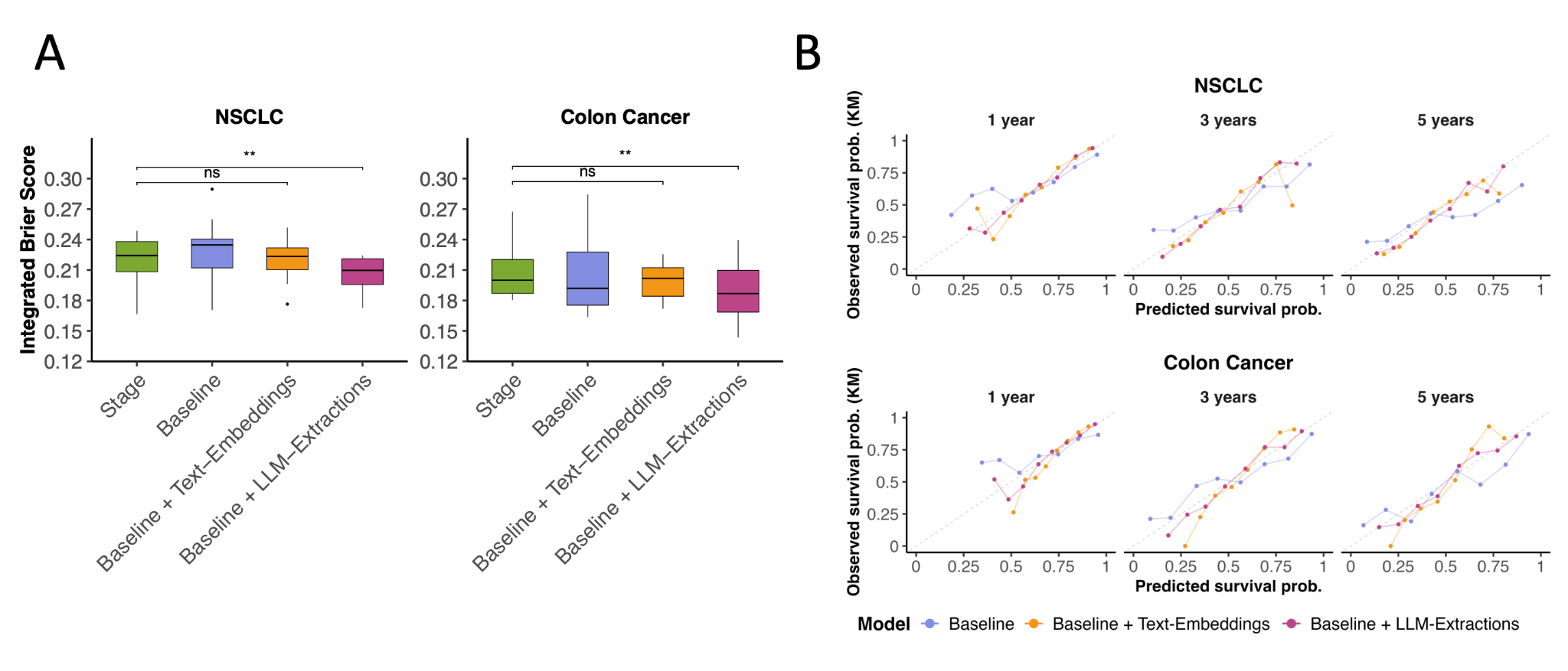


**S10 Fig: Integrated Brier Score and calibration of survival models in NSCLC and colon cancer.** **A:** Integrated Brier Score (IBS) for each model, computed using 10-fold cross-validation. For each fold, the Brier Score was evaluated at 1, 3, and 5 years using predicted survival probabilities, then integrated over these timepoints. Censoring was accounted for via inverse probability of censoring weighting (IPCW) estimated from the training fold. Boxplots show the distribution across folds. Pairwise comparisons against the Stage-only model use the paired Wilcoxon signed-rank test (ns: not significant; **: p<0.01). **B:** Calibration curves for NSCLC (top) and colon cancer (bottom) at 1, 3, and 5 years, comparing predicted versus Kaplan-Meier-observed survival probabilities across equidistant bins of the predicted probability axis.
