# Supplementary material for: Large language models enable prognostic stratification of cancer patients using real-world clinical notes
Source: PLOS Digit Health. 2026 Jul 8;5(7):e0001546. doi: 10.1371/journal.pdig.0001546 (PMC13345263; doi:10.1371/journal.pdig.0001546)
Supplement: S11 Fig — (DOCX) [file pdig.0001546.s012.docx]

**
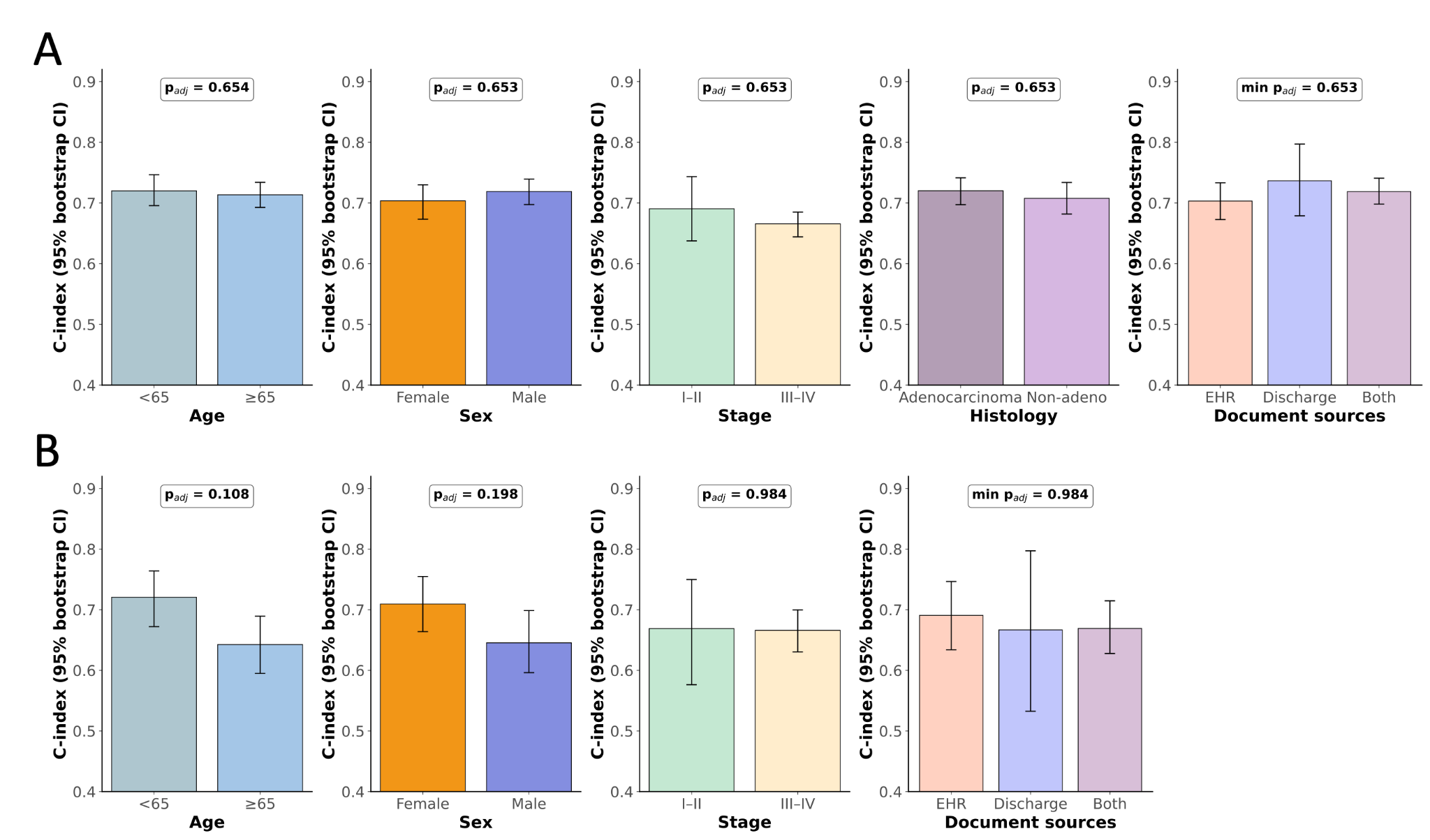
**

**S11 Fig: Subgroup analysis of model discrimination in NSCLC and colon cancer cohorts. A:** C-index of the Random Survival Forest trained on baseline clinical variables combined with LLM-extracted features, computed separately within patient subgroups of the NSCLC cohort. Bars show the C-index within each subgroup level; error bars indicate 95% bootstrap confidence intervals (1,000 resamples). Subgroups are defined by age (<65 vs ≥65 years), sex, stage (I–II vs III–IV), histology (adenocarcinoma vs non-adenocarcinoma), and available document sources (EHR notes only, discharge reports only, or both). Reported p-values were obtained by bootstrap tests of pairwise C-index differences, FDR-corrected across all subgroup comparisons. For subgroups with more than two levels, the minimum adjusted p-value across pairwise comparisons is shown (min p_adj). **B:** Identical analysis in the colon cancer cohort, stratified by age, sex, stage, and available document sources (histology subgroups not applicable).
