# Supplementary material for: Large language models enable prognostic stratification of cancer patients using real-world clinical notes
Source: PLOS Digit Health. 2026 Jul 8;5(7):e0001546. doi: 10.1371/journal.pdig.0001546 (PMC13345263; doi:10.1371/journal.pdig.0001546)
Supplement: S1 Table — (DOCX) [file pdig.0001546.s014.docx]

**S1 Table: Validation of LLM-extracted patient condition indicators in the NSCLC cohort.** Precision, recall, F1 score, and accuracy of LLM-extracted patient condition indicators (PCIs), evaluated against physician annotations on 50 randomly sampled patients. The macro average across all PCIs is shown in the bottom row. Cells with fewer than 4 expert-positive cases are reported as n.a. due to unstable metric estimates at low positive counts.

| **Patient condition indicator** | Precision | Recall | F1 | Accuracy |
| --- | --- | --- | --- | --- |
| B-Symptoms | 0.80 | 0.50 | 0.62 | 0.90 |
| Pain | 0.42 | 0.62 | 0.50 | 0.80 |
| Abnormal Physical Examination | 0.94 | 0.89 | 0.92 | 0.94 |
| High-risk status | 0.76 | 0.93 | 0.84 | 0.80 |
| Mobility Impairment | 1.00 | 0.50 | 0.67 | 0.96 |
| Dyspnea | 0.65 | 0.85 | 0.73 | 0.84 |
| Complicated DC | 0.74 | 0.89 | 0.81 | 0.84 |
| *Macro average* | 0.76 | 0.74 | 0.73 | 0.87 |
