# Supplementary material for: Large language models enable prognostic stratification of cancer patients using real-world clinical notes
Source: PLOS Digit Health. 2026 Jul 8;5(7):e0001546. doi: 10.1371/journal.pdig.0001546 (PMC13345263; doi:10.1371/journal.pdig.0001546)
Supplement: S3 Table — (DOCX) [file pdig.0001546.s016.docx]

**S3 Table: LLM extraction accuracy by patient subgroup, NSCLC cohort.** Per-subgroup F1 scores comparing LLM-extracted patient condition indicators (PCIs) to expert annotations on 50 annotated NSCLC patients, stratified by subgroups. The "Overall" row reports performance across all 50 annotated patients. For each (subgroup x PCI) cell, the F1 score is shown as well as the number of expert-positive cases out of the total annotated patients in that subgroup in parentheses. Cells with fewer than 4 expert-positive cases are reported as n.a. due to unstable F1 estimates at low positive counts.

| **Subgroup** | **Level** | **B-symp.** | **Pain** | **Abn. PE** | **High risk** | **Mobility** | **Dysp.** | **Compl. DC** |
| --- | --- | --- | --- | --- | --- | --- | --- | --- |
| Overall |  | 0.62  (8/50) | 0.5  (8/50) | 0.92  (19/50) | 0.84  (28/50) | 0.67  (4/50) | 0.73  (13/50) | 0.81  (19/50) |
| Age | <65 | n.a.  (3/22) | n.a.  (3/22) | 0.93  (7/22) | 0.93  (15/22) | n.a.  (2/22) | 0.77  (6/22) | 0.78  (10/22) |
|  | ≥65 | 0.57  (5/28) | 0.55  (5/28) | 0.91  (12/28) | 0.75  (13/28) | n.a.  (2/28) | 0.71  (7/28) | 0.84  (9/28) |
| Sex | Female | n.a.  (1/19) | n.a.  (3/19) | 0.91  (5/19) | 0.82  (9/19) | n.a.  (2/19) | n.a.  (2/19) | 0.93  (7/19) |
|  | Male | 0.73  (7/31) | 0.62  (5/31) | 0.92  (14/31) | 0.85  (19/31) | n.a.  (2/31) | 0.78  (11/31) | 0.74  (12/31) |
| Stage | I–II | 0.4  (4/21) | n.a.  (3/21) | 0.93  (8/21) | 0.8  (11/21) | n.a.  (2/21) | 0.73  (4/21) | 0.71  (7/21) |
|  | III–IV | 0.75  (4/29) | 0.77  (5/29) | 0.91  (11/29) | 0.86  (17/29) | n.a.  (2/29) | 0.74  (9/29) | 0.86  (12/29) |
| Histology | Adenocarcinoma | n.a.  (1/27) | 0.55  (6/27) | 0.92  (7/27) | 0.76  (12/27) | n.a.  (2/27) | 0.5  (4/27) | 0.7  (9/27) |
|  | Non-adeno | 0.6  (7/23) | n.a.  (2/23) | 0.92  (12/23) | 0.91  (16/23) | n.a.  (2/23) | 0.82  (9/23) | 0.91  (10/23) |

B-symp.=B-symptoms, Abn. PE=abnormal physical examination, High risk=high-risk status, Mobility=mobility impairment, Dysp.=dyspnea, Compl. DC=complicated disease course.
