# Supplementary material for: Large language models enable prognostic stratification of cancer patients using real-world clinical notes
Source: PLOS Digit Health. 2026 Jul 8;5(7):e0001546. doi: 10.1371/journal.pdig.0001546 (PMC13345263; doi:10.1371/journal.pdig.0001546)
Supplement: S4 Table — (DOCX) [file pdig.0001546.s017.docx]

**S4 Table: LLM extraction accuracy by patient subgroup, colon cancer cohort.** Per-subgroup F1 scores comparing LLM-extracted patient condition indicators (PCIs) to expert annotations on 50 annotated colon cancer patients, stratified by subgroups. The "Overall" row reports performance across all 50 annotated patients. For each (subgroup x PCI) cell, the F1 score is shown as well as the number of expert-positive cases out of the total annotated patients in that subgroup in parentheses. Cells with fewer than 4 expert-positive cases are reported as n.a. due to unstable F1 estimates at low positive counts.

| **Subgroup** | **Level** | **B-symp.** | **Pain** | **Abn. PE** | **High risk** | **Mobility** | **Dysp.** | **Compl. DC** |
| --- | --- | --- | --- | --- | --- | --- | --- | --- |
| Overall |  | n.a.  (0/50) | 0.86  (18/50) | 1.0  (13/50) | 0.82  (23/50) | 0.8  (5/50) | n.a.  (2/50) | 0.81  (22/50) |
| Age | <65 | n.a.  (0/27) | 0.87  (12/27) | 1.0  (8/27) | 0.78  (11/27) | n.a.  (3/27) | n.a.  (1/27) | 0.8  (10/27) |
|  | ≥65 | n.a.  (0/23) | 0.83  (6/23) | 1.0  (5/23) | 0.85  (12/23) | n.a.  (2/23) | n.a.  (1/23) | 0.82  (12/23) |
| Sex | Female | n.a.  (0/22) | 0.89  (9/22) | 1.0  (7/22) | 0.92  (12/22) | n.a.  (3/22) | n.a.  (2/22) | 0.89  (10/22) |
|  | Male | n.a.  (0/28) | 0.82  (9/28) | 1.0  (6/28) | 0.72  (11/28) | n.a.  (2/28) | n.a.  (0/28) | 0.75  (12/28) |
| Stage | I–II | n.a.  (0/23) | 0.88  (7/23) | 1.0  (5/23) | 0.82  (7/23) | n.a.  (3/23) | n.a.  (2/23) | 0.4  (7/23) |
|  | III–IV | n.a.  (0/27) | 0.84  (11/27) | 1.0  (8/27) | 0.81  (16/27) | n.a.  (2/27) | n.a.  (0/27) | 0.94  (15/27) |

B-symp.=B-symptoms, Abn. PE=abnormal physical examination, High risk=high-risk status, Mobility=mobility impairment, Dysp.=dyspnea, Compl. DC=complicated disease course.
