# Supplementary material for: Large language models enable prognostic stratification of cancer patients using real-world clinical notes
Source: PLOS Digit Health. 2026 Jul 8;5(7):e0001546. doi: 10.1371/journal.pdig.0001546 (PMC13345263; doi:10.1371/journal.pdig.0001546)
Supplement: S5 Table — (DOCX) [file pdig.0001546.s018.docx]

**S5 Table: Results of univariate Cox proportional-hazards models evaluating the association between LLM-extracted covariates and overall survival.** For each covariate, the hazard ratio (HR), 95 % confidence interval (CI) and p-value are reported. Structured EHR data comprises all fields originally available in structured format, whereas LLM-inferred variables are those derived by the model from unstructured medical documentation.

|  | **NSCLC Univariate analysis** | | **Colon Cancer Univariate analysis** | |
| --- | --- | --- | --- | --- |
| **Structured EHR Data** | **HR (95% CI)** | **P value** | **HR (95% CI)** | **P value** |
| Age at Treatment (per 1 SD) | 1.03 (0.97-1.1) | 0.301 | 1.24 (1.1-1.4) | **<0.001** |
| Stage II vs I | 1.53 (1.08-2.15) | **0.016** | 0.62 (0.31-1.22) | 0.163 |
| Stage III vs I | 2.34 (1.80-3.04) | **<0.001** | 0.84 (0.45-1.55) | 0.570 |
| Stage IV vs I | 4.10 (3.26-5.14) | **<0.001** | 2.61 (1.57-4.33) | **<0.001** |
| Sex (male) | 1.34 (1.18-1.53) | **<0.001** | 0.88 (0.7-1.11) | 0.278 |
| Adenosquamous vs Adenocarcinoma | 1.09 (0.8-1.47) | 0.599 |  |  |
| Large Cell vs Adenocarcinoma | 1.05 (0.77-1.43) | 0.767 |  |  |
| Squamous cell vs Adenocarcinoma | 1.07 (0.93-1.23) | 0.354 |  |  |
| **LLM-Inferred Variables** |  |  |  |  |
| High-Risk Status | 1.41 (1.21-1.64) | **<0.001** | 1.85 (1.42-2.42) | **<0.001** |
| Abnormal Physical Examination | 2.01 (1.78-2.28) | **<0.001** | 1.79 (1.43-2.25) | **<0.001** |
| Dyspnea | 1.56 (1.38-1.77) | **<0.001** | 1.61 (1.14-2.27) | **0.007** |
| Complicated Disease Course | 2.19 (1.92-2.5) | **<0.001** | 1.92 (1.5-2.45) | **<0.001** |
| B-Symptoms | 1.99 (1.72-2.3) | **<0.001** | 2.11 (1.63-2.74) | **<0.001** |
| Pain | 1.62 (1.43-1.84) | **<0.001** | 1.28 (1.02-1.61) | **0.031** |
| Mobility Impairment | 2.64 (2.25-3.09) | **<0.001** | 2.4 (1.8-3.19) | **<0.001** |
