# Supplementary material for: Large language models enable prognostic stratification of cancer patients using real-world clinical notes
Source: PLOS Digit Health. 2026 Jul 8;5(7):e0001546. doi: 10.1371/journal.pdig.0001546 (PMC13345263; doi:10.1371/journal.pdig.0001546)
Supplement: S6 Table — (DOCX) [file pdig.0001546.s019.docx]

**S6 Table: Results of univariate and multivariate Cox proportional hazards models for the NSCLC cohort evaluating the association between LLM-inferred scores and overall survival.** In the univariate analysis, each feature was tested individually. For each covariate, the hazard ratio (HR), 95 % confidence interval (CI) and p-value are reported. Structured EHR data comprises all fields originally available in structured format, whereas LLM-inferred variables are those derived by the model from unstructured medical documentation.

|  | **Univariate analysis** | | **Multivariate analysis** | |
| --- | --- | --- | --- | --- |
| **Structured EHR Data** | **HR (95% CI)** | **P value** | **HR (95% CI)** | **P value** |
| Age at Treatment (per 1 SD) | 1.03 (0.97-1.1) | 0.301 | 1.1 (1.03-1.17) | **0.004** |
| Stage II vs I | 1.53 (1.08-2.15) | **0.016** | 1.43 (1.01-2.02) | **0.042** |
| Stage III vs I | 2.34 (1.8-3.04) | **<0.001** | 2.47 (1.9-3.21) | **<0.001** |
| Stage IV vs I | 4.1 (3.26-5.14) | **<0.001** | 4.51 (3.57-5.69) | **<0.001** |
| Sex (male) | 1.34 (1.18-1.53) | **<0.001** | 1.33 (1.17-1.52) | **<0.001** |
| **LLM-Inferred Variables** |  |  |  |  |
| Physical Condition Score (per 1 SD) | 0.82 (0.78-0.86) | **<0.001** | 0.79 (0.74-0.84) | **<0.001** |
| Survival Score (per 1 SD) | 0.81 (0.77-0.86) | **<0.001** | 0.97 (0.9-1.05) | 0.471 |
