# Supplementary material for: Large language models enable prognostic stratification of cancer patients using real-world clinical notes
Source: PLOS Digit Health. 2026 Jul 8;5(7):e0001546. doi: 10.1371/journal.pdig.0001546 (PMC13345263; doi:10.1371/journal.pdig.0001546)
Supplement: S7 Table — (DOCX) [file pdig.0001546.s020.docx]

**S7 Table: Results of univariate and multivariate Cox proportional hazards models for the colon cancer cohort evaluating the association between LLM-inferred scores and overall survival.** In the univariate analysis, each feature was tested individually. For each covariate, the hazard ratio (HR), 95 % confidence interval (CI) and p-value are reported. Structured EHR data comprises all fields originally available in structured format, whereas LLM-inferred variables are those derived by the model from unstructured medical documentation.

|  | **Univariate analysis** | | **Multivariate analysis** | |
| --- | --- | --- | --- | --- |
| **Structured EHR Data** | **HR (95% CI)** | **P value** | **HR (95% CI)** | **P value** |
| Age at Treatment (per 1 SD) | 1.24 (1.1-1.4) | **<0.001** | 1.42 (1.24-1.62) | **<0.001** |
| Stage II vs I | 0.62 (0.31-1.22) | 0.163 | 0.52 (0.26-1.04) | 0.066 |
| Stage III vs I | 0.84 (0.45-1.55) | 0.570 | 0.78 (0.42-1.47) | 0.448 |
| Stage IV vs I | 2.61 (1.57-4.33) | **<0.001** | 2.71 (1.6-4.58) | **<0.001** |
| Sex (male) | 0.88 (0.7-1.11) | 0.278 | 0.87 (0.69-1.09) | 0.234 |
| **LLM-Inferred Variables** |  |  |  |  |
| Physical Condition Score (per 1 SD) | 0.79 (0.72-0.86) | **<0.001** | 0.86 (0.75-0.98) | **0.021** |
| Survival Score (per 1 SD) | 0.69 (0.62-0.77) | **<0.001** | 0.81 (0.7-0.95) | **0.010** |
